# Systematic Analysis of Pigeons’ Discrimination of Pixelated Stimuli: A Hierarchical Pattern Recognition System Is Not Identifiable

**DOI:** 10.1038/s41598-019-50212-1

**Published:** 2019-09-26

**Authors:** Juan D. Delius, Julia A. M. Delius

**Affiliations:** 1 Experimental Psychology, University of Konstanz, 78457 Konstanz, Germany; 2 Center for Lifespan Psychology, Max Planck Institute for Human Development, 14195 Berlin, Germany

**Keywords:** Pattern vision, Psychology

## Abstract

Pigeons learned to discriminate two different patterns displayed with miniature light-emitting diode arrays. They were then tested with 84 interspersed, non-reinforced degraded pattern pairs. Choices ranged between 100% and 50% for one or other of the patterns. Stimuli consisting of few pixels yielded low choice scores whereas those consisting of many pixels yielded a broad range of scores. Those patterns with a high number of pixels coinciding with those of the rewarded training stimulus were preferred and those with a high number of pixels coinciding with the non-rewarded training pattern were avoided; a discrimination index based on this correlated 0.74 with the pattern choices. Pixels common to both training patterns had a minimal influence. A pixel-by-pixel analysis revealed that eight pixels of one pattern and six pixels of the other pattern played a prominent role in the pigeons’ choices. These pixels were disposed in four and two clusters of neighbouring locations. A summary index calculated on this basis still only yielded a weak 0.73 correlation. The individual pigeons’ data furthermore showed that these clusters were a mere averaging mirage. The pigeons’ performance depends on deep learning in a midbrain-based multimillion synapse neuronal network. Pixelated visual patterns should be helpful when simulating perception of patterns with artificial networks.

## Introduction

With so many laboratory pigeons in the contemporary behavioural literature responding to complex images on touchscreens, the fact that their wild ancestors’ pecking behaviour mainly evolved as a plain manoeuvre to acquire food has almost been forgotten. The response involves a straightforward grasping of nourishing grains in preference to grit particles and other background. Furthermore pigeons as a species, and even as individuals, are known to have pronounced likes for only certain kinds of seeds^1–6^. Pigeons are furthermore inclined to avoid grains slightly stained by moulds or bored by weevils and, at a more global level, to be selective about the varieties of ears of grain (wheat, oats, barley, rye) on which they often feed in the wild (J. D. Delius, unpublished observations, 1986).

In 1972, impressed by the variegated but idiosyncratic food preferences of an individual whorl-crowned piebald, free-ranging city pigeon at “Sweaty Betty’s” legendary fish and chip shop, it occurred to some psychology students who were inspired by a preceding tachistoscopic practical on redundancy reduction in pattern recognition in human subjects, and the senior author J.D.D., then at Durham University, UK, to confront mildly hungry pigeons with 3 ml heaps of a mixture of some 20 seeds with a variety of some 20 stone, ceramic, glass, metal, and plastic particles, all sieved to be of a similar size range of 1.5 to 3 mm in diameter. We were surprised at the speed and accuracy with which the pigeons would preferentially and individually select and consume only certain kinds of seeds –and occasionally certain types of grit– out of the heaps of this mixture that we successively offered them. However, the selection process was so fast that it could not be satisfactorily resolved with the video-slowing equipment available. Nevertheless, we could ascertain that the six pigeons significantly preferred to first peck at a scattered 3 cm diameter patch of 12 mixed small grains rather than at a concentrated 1 cm diameter conical heap of 12 mixed small grains presented as pairs side by side, sides randomized, on a 12 × 6 cm flat hardboard platform (20 trials per pigeon, average 17.8 scattered patch choices, binomial test, p < 0.01; cf.^7^).

**Figure 1:**
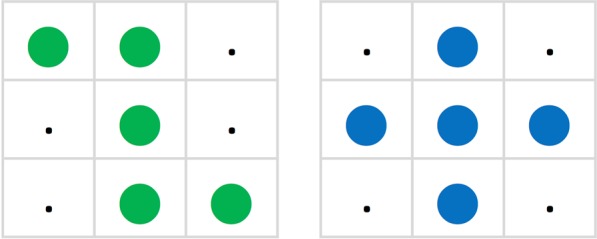
Pair of z and +, initially equi-coloured milo-grain patterns.

We then resorted to a different approach using a heavy wooden block that was successively hung onto four individual pigeon cages, replacing their standard food troughs. This platform was provided with two milled parallel grooves about 3 cm apart. Through these grooves the experimenters could simultaneously push forward two white pinewood strips, 2.5 cm wide, 0.3 cm thick, 10 cm long, so that they both protruded 2.5 cm into the cage. This brought two fields of 15 × 15 mm, each with a set of 3 × 3 punch-stamped hemispherical depressions at the ends of the strips into the pigeons’ view and access. These two fields were each filled with five brown milo seeds (*Sorghum bicolor*) snugly occupying the two nine-hollow fields according to patterns resembling a z or a + (Fig. 1). The pigeons were prevented from seeing the experimenter by a vertical white-clad hardboard screen with a rectangular 6 × 12 cm port covered with grey nylon gauze serving as a one-way window. Through it one could view the brightly illuminated milo-seed fields and record the first peck-grasp response of the pigeons towards the two different food offerings. In successive trials, the wooden strips bearing the two patterns would alternate in their right and left positions according to quasi-random^8^ sequences. For two pigeons, the + pattern consisted of five milo seeds firmly but invisibly glued to the wooden strip with a small drop of epoxy adhesive, whilst the five milo seeds of the z pattern would always lie loose in the hollows; for the other two pigeons the arrangement was the precise converse. The birds were kept food deprived at 85% of their ad-lib weight. On each trial they were given sufficient time to consume all the loose milo seeds. We had routinely prepared 10 glued seed pattern whitewood strips and 10 pre-loaded loose seed pattern strips from which we picked one pair at random before each trial. Over successive trials the pigeons learned to preferentially peck at the loose milo pattern first. After 12 twice-daily sessions, each consisting of 30 trials, all 4 pigeons yielded significant (binomial tests, p < 0.05) evidence of preferring the loose pattern on the last session ( > 20 correct, < 10 incorrect choices, achieving at least 70% correct choices). But we also observed that the pigeons would occasionally be quite persistent in trying to pry the grains off the glued pattern. This response is of course natural to them, for example when feeding on corn on the cob or bread crusts. Following this, we ran every second session with 6 extra test trials interspersed among the 24 normal trials. These test trials involved pairs of variously degraded patterns consisting of 1, 2, 3, or 4 glued milo seeds. Overall the choice scores of these degraded z and + -like patterns rose with the number of seeds involved, suggesting that the pigeons assessed them as global constellations. But we also obtained some, though weaker evidence that the birds could distinguish the patterns by only attending to one or the other of the four truly differential milo seed locations. We could relate this to the impression that as the discrimination training progressed, the pigeons had come to preferentially direct their first pecks to either of the two truly distinctive seed locations of the reinforced z and + pattern.

We next differentiated the two training patterns by employing white variety milo seeds coloured either red or blue with food dye. Within four further sessions, the discrimination by all six pigeons shot up to a nearly perfect 98% correct choices. When, however, the pigeons were afterwards switched to discriminate the z and + patterns, each assembled with a mix of red and blue seeds, the pigeons tended to preferentially peck at the previously positive coloured seeds nearly regardless of which pattern they conformed. The pigeons now proved quite recalcitrant to retraining to choose one or the other of the bicoloured z and + patterns: Colour, an attribute of the individual seeds, seemed to have taken a strong precedence over the global shapes as a discriminative cue.

But the seed-by-seed discrimination was still a very fast process that we could not properly track. As we now know, pigeons customarily peck grains out of a small pattern, or a small heap, at the rate of about 3 pecks per second^9–11^. For adequate resolution, their seed (and grit) choice behaviour has to be high-speed video recorded and slow-speed reviewed, a procedure that unless automated, is too time- and labour-consuming for routine use. Therefore, although these preliminary experiments led to several suggestive observations, they only yielded a few sufficiently verified results concerning the selective pecking. Later, however, our laboratory regularly employed a derived but much simplified grit–grain discrimination procedure to assess the visual competences of pigeons in various contexts^12–14^ (see also^15^, for related experiments on neonate chickens).

As already noted, studies involving pigeons’ learned discrimination of complex visual stimuli other than food have of course a long tradition, and many of these have contributed importantly to the field of comparative cognition. They have mainly been concerned with the evaluation and memorization of such stimuli. Fewer studies have focused on the underlying perceptual processing of the elements composing such stimuli. Most of the stimuli used have been of an either too simple, or conversely, too complex nature to make a detailed analysis of perceptual processing feasible. Where such analysis has been attempted, it has mainly been guided by notions derived from human perception studies^16–21^, but see^22^. That it may be misguided to assume that birds use a quite human-like perceptual information processing has been best established for colour vision, which is at least tetra-chromatic in birds rather than only tri-chromatic^23,24^. Divergences may not least come about because avian vision is predominantly based on tectal rather than cortical information processing^25–29^. Traditionally the midbrain of vertebrates, and especially the optic tecta of birds, have not been considered as being particularly neuroplastic. However, the profuse presence of the synaptic transmitter glutamate as well as its matching NMDA and AMPA postsynaptic receptors in avian tecta and post-tecta and even retinae^30–33^ suggests that it could be the substrate of multifarious learning processes.

**Figure 2:**
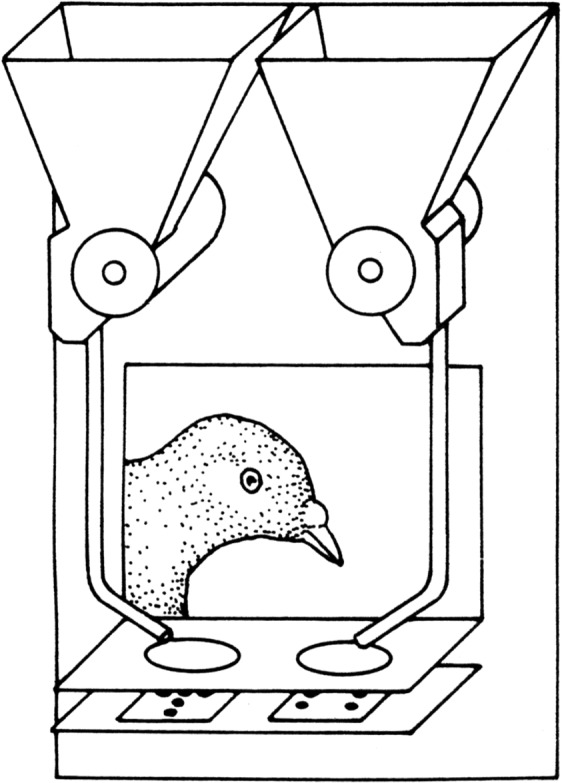
Conditioning platform furnished with light-emitting diode (LED) matrices.

Based on the wood-block devices described above, our Konstanz lab introduced a somewhat novel instrumental conditioning device for pigeons in 1995^34^ (Fig. 2), these being addressed as habitual ground-feeders. It is a ‘Skinner’ platform equipped with two separate 5 × 7 light-emitting diode (LED) matrices presenting stimuli under two transparent, horizontal pecking keys that could be hung on the pigeons’ home cages, replacing their regular feeding troughs. The matrices served to generate discretely pixelated, simple visual patterns which the pigeons could be taught to discriminate for grain reward that was automatically delivered on the keys. Several experiments carried out with these appliances have collaterally suggested that pigeons might attend as much to locally lit LEDs as to holistic patterns encompassing larger parts of the matrices^35–37^. That pigeons may sometimes be prone to preferentially attend to picture details (local features) rather than the whole picture (global patterns) has since also become apparent in other studies using complex visual stimuli^20,21,38–40^. The present experiment was based on the expectation that the LED matrix pattern discrimination would have similarities with the foraging operations on grains we had attempted to study in our early Durham experiments (see above), and would cast light on how the pigeons’ visual system composes visual patterns out of visual elements. For an interesting comparison, see also Bachmann (2015)^41^, who provides a general introduction to the (human) perception of pixelated images.

**Figure 3:**
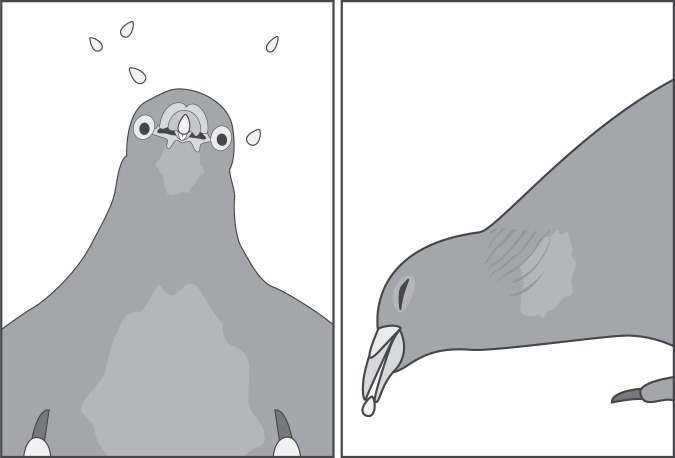
Pigeon examining scattered grains with convergent eyes prior to pecking at a grain just below its beak tip (left) and then grasping a grain with half-closed eyes (right).

It is opportune to remind readers that pigeons usually examine grains and grit from 2 to 3 successive head stop positions involving convergent eye fixations from 10 to 5 cm away. They then proceed to beak-grasp the chosen item with the gape opened to slightly more than grain size and a forward-down head thrust with the eyes half closed to a slit, this closure protecting the cornea from backscatter but probably also serving to increase depth of focus by an aperture reduction (Fig. 3). Tactile-gustatory-auditory evaluation by the pigeon then leads to the item either being swallowed or dropped. When dealing with heaps of grains and grit pigeons often also use sideward closed-beak swishing movements to scatter the items^10,42–44^.

## Methods

### Subjects and apparatus

Six adult domestic pigeons (*Columba livia*), bred by the University of Konstanz Animal Facility out of local homing stock, were used. They were kept in individual 40 × 45 × 35 cm wire grid cages located in a well-ventilated and mainly naturally, partly artificially illuminated (12 h on/12 h off) room and were maintained at about 85% of their normal weight throughout the experiment. All the experimental treatments described in this paper received prior approval according to the German Tierschutzgesetz [Animal Welfare Law] by the district’s Tierschutzkommission [Animal Protection Committee], Freiburg-im-Breisgau, Germany, and all methods were performed in accordance with the relevant guidelines and regulations.

Horizontal conditioning platforms controlled by a Dell personal computer equipped with a digital interface were used^34^. These were attached to the pigeons’ home cages replacing their standard feeding troughs. Each platform (6 × 9 cm) incorporated two side-by-side transparent pecking keys (diameter 2.5 cm, centres 5 cm apart). Two red LED matrices (5 × 7 diodes, 12 × 17 mm) placed under these keys served to present pixelated visual patterns. The LEDs were embedded within a slightly frosted plastic plate that diffused their optical outlines. Two overhead solenoid feeders could deliver rewards consisting of a few grains of millet onto the dish-shaped keys (cf. Fig. 2).

We were unsuccessful in an attempt to replace the whole-display pecking keys with miniature touch-screens allowing an individual pixel-by-pixel peck detection, finding that they much distracted the pigeons from their pattern choosing behaviour: they were inclined to peck at the lateral infrared LEDs that were part of the detection device. Furthermore, they also appeared to be disturbed by their beak tips lighting-up as they traversed the horizontal infrared beams^42,45,46^. We were thus limited to the evaluation of observational and/or of correlational data as to the role of the various pixel locations for the pattern discriminations. Observations during tests involving patterns with few, scattered lit pixels (see later stimulus figures for examples) showed that the pigeons would often persist in pecking at a singular pixel for runs of trials at a time.

### Training procedure

**Figure 4:**
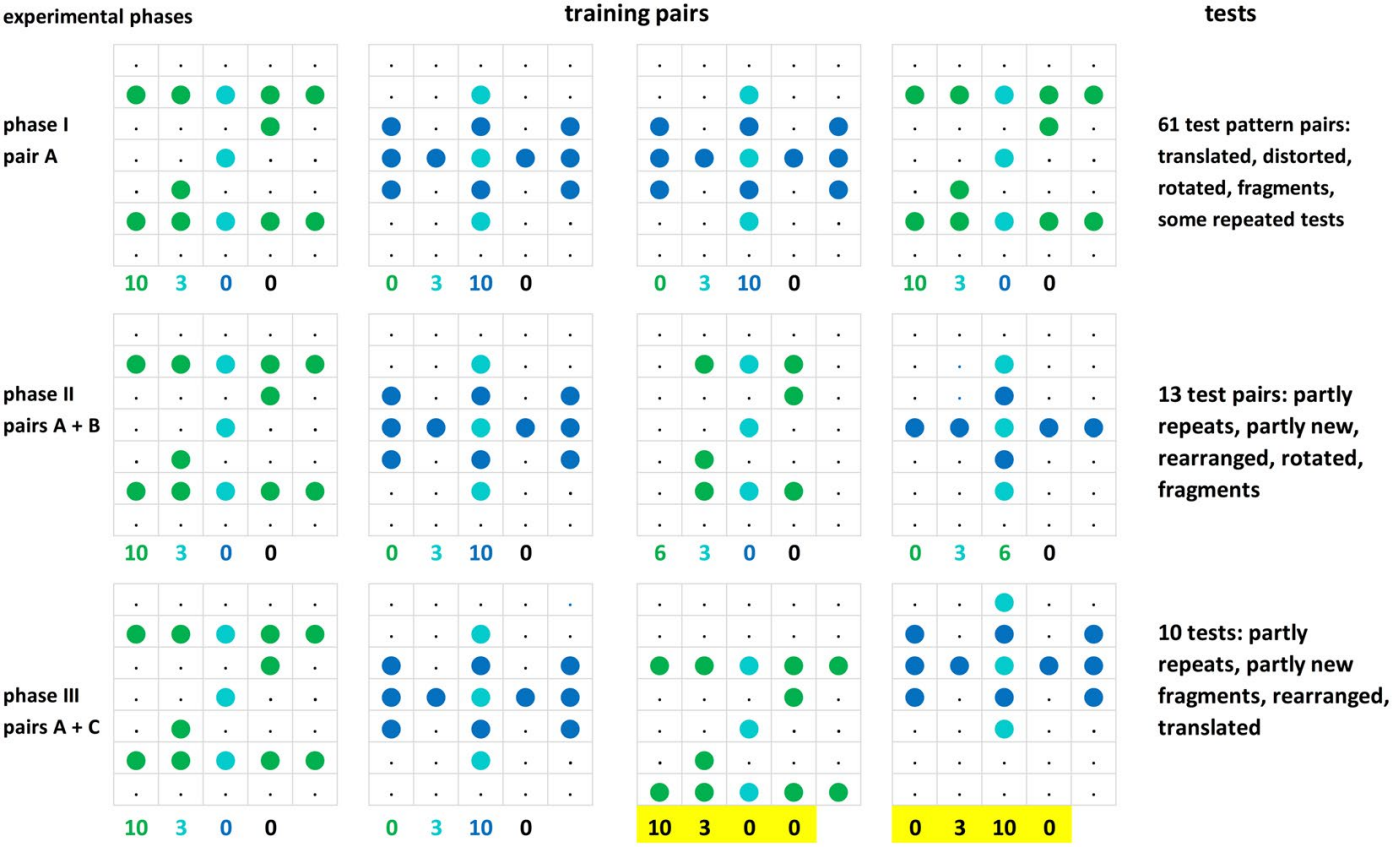
Experimental phases I, II, and III with training patterns A, B, and C, and summaries of testing procedures. Under the patterns: counts of z-specific (green), common to zh (turqoise), h-specific (blue), and neutral (n) (unshaded, unlit) pixels. The black counts highlighted in yellow are corrected as explained in the later *Discrimination measure* and *Translocated and compound patterns* sections. Note that the pigeons saw all of the patterns formed by red-lit diodes; the colourations shown in the figures solely serve to identify their component pixels according to the schema shown in Fig. 5.

The pigeons were first shaped to peck the keys. This involved daily sessions of 100 trials each. Each trial within these sessions began with a 20 s pause (or inter-trial-interval, ITI). A stimulus with all 35 pixels lit was then presented for 8 s randomly under either the right or the left key, the other key remaining unlit. A peck to the illuminated key delivered an immediate reward onto this key followed by a 2 s feeding time. If the pigeon did not peck, the reward was automatically issued at the end of the stimulus presentation. As soon as 80% of the trials yielded a peck, these latter free rewards were discontinued and rewards only delivered when the pigeons pecked the key while it was stimulus-pattern lit; pecks at the unlit keys had no scheduled consequences. When the subjects had emitted 80 or more such instrumental responses within a session, they proceeded to the discrimination training stage of phase I. They were taught to discriminate patterns A (Fig. 4), one resembling the letter Z (Z* for short) and the other resembling a Viking cross (H* for short), each composed of 13 lit LEDs (= lit pixels) and 22 unlit LEDs (= unlit pixels) using a simultaneous stimulus presentation, discrete trial procedure. For three randomly selected Z^+^H^−^ pigeons, the pattern Z* was correct (i.e., choices thereof were rewarded), whereas for the other three H^+^Z^-^ pigeons, the pattern H* was correct (i.e., choices thereof were rewarded). The daily training sessions, except for weekends, consisted of 3 blocks of 50 trials each. Following the now routine 2 s ITI, a trial began with the simultaneous presentation of the two discriminative stimuli under the keys. The right or left allocation of the positive (correct) and negative (incorrect) patterns over successive trials was quasi-random^47^.

With a randomized probability of 4 out of 5 (partial reinforcement p = 0.80) according to another quasi-random^47^ sequence, 6 consecutive pecks delivered to the key showing the correct stimulus yielded a grain reward on the key, followed by a 2 s feeding time with an all-lit matrix below the key. With the same 0.80 probability, 6 consecutive pecks to the key displaying the incorrect stimulus yielded a penalty consisting of a 2 s time-out with all-dark matrices. The reaction times after stimulus onset were not routinely recorded but in a one-session probe across the 6 pigeons towards the end of their first training phase we obtained a mean of 0.63 ± 0.32 s to the first peck, regardless of whether it was correct or incorrect. The time to the final sixth peck reached the mean of 2.01 ± 0.64 s. Switching from pecking one key to pecking the other one within a trial –once up to maximally thrice– was a rare event, only occurring in 5.7% of the recorded trials. Occasional test trials, though not these switch trials in particular, were associated with noticeably longer reaction times –but pigeons are generally known to respond predominantly reaction-time invariantly while being quite error-rate variant at same time. The routine 2 s ITI with dark matrices preceded the next trial. The thus either-way reinforced training patterns will henceforth be designated with Z* and H*. Trials ending in penalty were followed by a repeat trial with the same stimulus pair. This correction procedure ended when the pigeon chose the correct stimulus. The choices produced during the correction trials were not included in the discrimination score calculations. The rarer non-reinforced choices were counted as correct or incorrect but led directly to the next 2 s ITI. When the pigeons had produced more than 90% correct non-correction trials on two successive sessions after 9–18 training sessions (mean = 12.3 sessions), they proceeded to the testing stage.

### Testing procedure

This involved daily sessions of 3 blocks of 50 trials each. The first and last training blocks of these sessions were exactly as described above. The middle block was composed of 30 training trials, also as described above, and 20 unreinforced test trials quasi-randomly^47^ inserted among them. These trials involved the presentation of novel pairs of pixel patterns consisting of 4–26 lit LEDs per pattern pair equally (or nearly equally) divided between the two stimuli forming the diverse pairs. Choices within the test trials –as before, six consecutive pecks to one or the other key– led to neither reward nor penalty but directly to the 2 s ITI before the next training or testing trial; there were obviously no correction trials in connection with these test trials. Within any given session only a single pair of test patterns was presented but over successive sessions many different stimulus pattern pairs were shown. Several pixel patterns were furthermore members of several different test pairs (see below). Some test pairs involved one or the other of the training patterns. The majority of the test patterns however differed to various degrees from the training patterns. Within each of the test pairs the test pattern that attracted the majority of the choices by the six pigeons –or within the separate teams by the three corresponding Z^+^H^−^ and H^+^Z^−^ pigeons– was deemed to be the pattern that the pigeons judged to be most similar to the rewarded training pattern(s). Choices of the corresponding unreinforced Z° or H° test patterns were counted as ‘correct,’ those of the other pattern as ‘incorrect.’ Each test session was followed by a regenerating, pure training session as described earlier. Whenever the performance of a pigeon fell below 90% correct on these refresher sessions –this happened only exceptionally–, the bird was retrained in more of such sessions until it re-attained the 90% correct criterion.

A total of 84 test sessions took place in three phases (Fig. 4). Phase I consisted of 61 test sessions involving the presentation of the standard training patterns as well as ever new test pattern pairs, even though some stimulus patterns recurred singly in these pairs. The last seven sessions (tests 54 to 61) of phase I involved the renewed presentation of the tests pairs already used earlier as a check for replicability. Phase II followed with the pigeons being trained with a half-and-half mix of trials involving the presentation of the original training pattern pairs A and a new pair of training patterns B. These were additional Z* and H* patterns, but with 4 pixels of each of the original patterns left unlit. They were expected to somewhat ‘broaden’ the representations that the pigeons might have formed of the preceding Z* and H* training patterns A and perhaps to make their generalization choices of subsequent Z°H° test patterns more ‘flexible.’ As soon as the pigeons had all re-attained the 90% correct choices criterion after 2–5 training sessions, they underwent 13 test sessions (tests 62–74) in which they continued to be trained with pattern pairs A and B but were additionally tested with test Z°H° pairs. Some of these had been used during the previous phase I, but some were novel. After phase II had ended and before testing phase III began, the pigeons were trained with the original training pair A and a new training pair C, in which the Z* and H* patterns were shifted respectively downwards ( = ‘south’) and upwards ( = ‘north’) by one row of pixels. This continued until all six pigeons had re-attained the 90% correct criterion after 4–7 training sessions. Again, the intention was to make the pigeon less individual-pixel and more pixel-pattern sensitive. Ten test sessions (tests 75–84) followed, in which the Z°H° test pairs were again partly repeats, but mainly novel. Training and testing sessions took place on a five days/week schedule with occasional days off and two breaks of three weeks. In all, the entire experiment took about seven months to complete.

The reader should keep in mind that any special spatial pixel co-occurrences ( = cross-correlations between pixel groups forming bars, obliques, vertices, crosses, etc.) within the complement of the 84 Z°H° test pairs, which might be thought as possibly influencing the later results, were in fact totally overshadowed by the far more frequent presentations (15:2 ratio, see above) of the Z*H* training pairs, where the co-occurrence correlation of the variously located z pixels and h pixels was a nearly perfect r = 1.00 throughout; albeit not completely because of the interspersed training with the fragmentary pair B in phase II (Fig. 4).

The 84 test pairs were obviously conformed from 84 H° and 84 Z° test patterns altogether, but because some of the patterns were used more than once, the effective numbers of distinct patterns were factually only 50 different Z° and 53 different H° patterns. Twenty-five different Z° stimulus patterns, respectively 30 different H° stimulus patterns were used only once. Seventeen further Z° and 17 further H° patterns were repeatedly used in two or three tests (see *Test replications* section). The remaining test pairs required were constructed with a further seven different Z° patterns and five different H° patterns used up to four times but avoiding repetition of the same pattern pairs. Care was taken that the repeated use of given patterns occurred in a well distributed manner within the test sequences (see *Test replications* section).

### Pattern pixel designations

**Figure 5:**
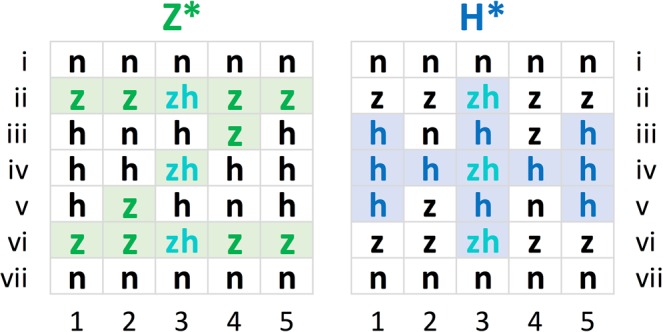
Pixel-by-pixel composition of the training stimulus patterns Z* and H* (from the mainly used set A), along with the pixel location designations. Green coloured letters indicate the lit z pixels unique to the Z* pattern, turquoise coloured letters indicate the lit zh pixels common to both patterns, and blue coloured letters indicate the lit h pixels unique to the H* pattern.

Figure 5 shows the column-by-row lit pixel z and h composition of the training Z* and H* patterns, phase I, each consisting of 10 lit z or h pixels plus 3 zh lit pixels in common, and 12 neutral (n) dark pixels in common. There were thus 12 n, 3 zh, 10 z, 10 h pixels = 35 × 2 total pixel locations under both keys that could be used afterwards in test trials. The uppermost and lowermost rows i and vii that were constituted by 10 of the 12 n pixel locations, however, were only lit in very few tests (see *Translocated and compound patterns* section below); they are therefore sometimes omitted in later figures and tables.

Note that during all training trials in phase I, besides the whole pattern being lit, any of the 10 z pixels, or alternatively any of the 10 h pixels being lit signalled a highly probable reward availability, or conversely, a certain non-availability of reward upon activation of the corresponding key for the Z^+^H^−^ and H^+^Z^−^ team pigeons (within-pattern pixel redundancy). The training Z* and H* patterns were thus predictive of reward and non-reward both at the global-pattern as well as the local-pixel levels. On the other hand, the darkness of any zh, z, or h pixel location under at least one key signalled the presence of a test pattern associated with the certain unavailability of any immediate food reward, though access to the presentation of a further training trial was linked to a very probable food reward. The darkness of any n pixel was uninformative about the prospect of reward, whereas any of them being lit was fully indicative of an absence of reward.

## Results

### Training

The pigeons learned to discriminate the Z* and H* training patterns to a criterion of no less than 90% correct responses within an average of 8 sessions (range 6–16 sessions, n = 6 pigeons). Afterwards, during phase I, we continued to record the percent correct choices on the training I pattern pair trials of the test sessions for each of the pigeons. Both the Z^+^H^−^ and H^+^Z^−^ groups very nearly always exceeded the 90% correct performance level on the training stimulus component across all 84 testing sessions, including also the later sessions of phase II and III. Only very occasionally –after less than 5% of these sessions– did one or the other pigeon require one or maximally two intercalated extra training sessions to re-attain this at least 90% correct criterion.

### Test replications

**Figure 6:**
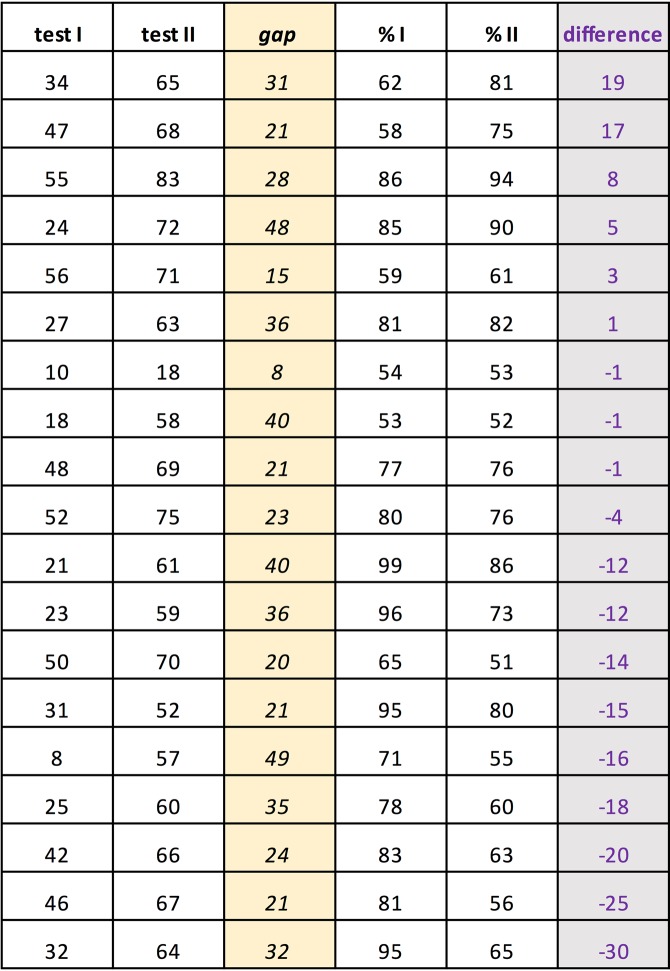
The 19 test replications (test I and test II) ordered according to the sign of the resulting percent choice differences from + 19 to −30 percent points (in violet, shaded grey) along with the corresponding test gaps (in italics, shaded yellow) from 8 to 49 sessions between the tests.

**Figure 7:**
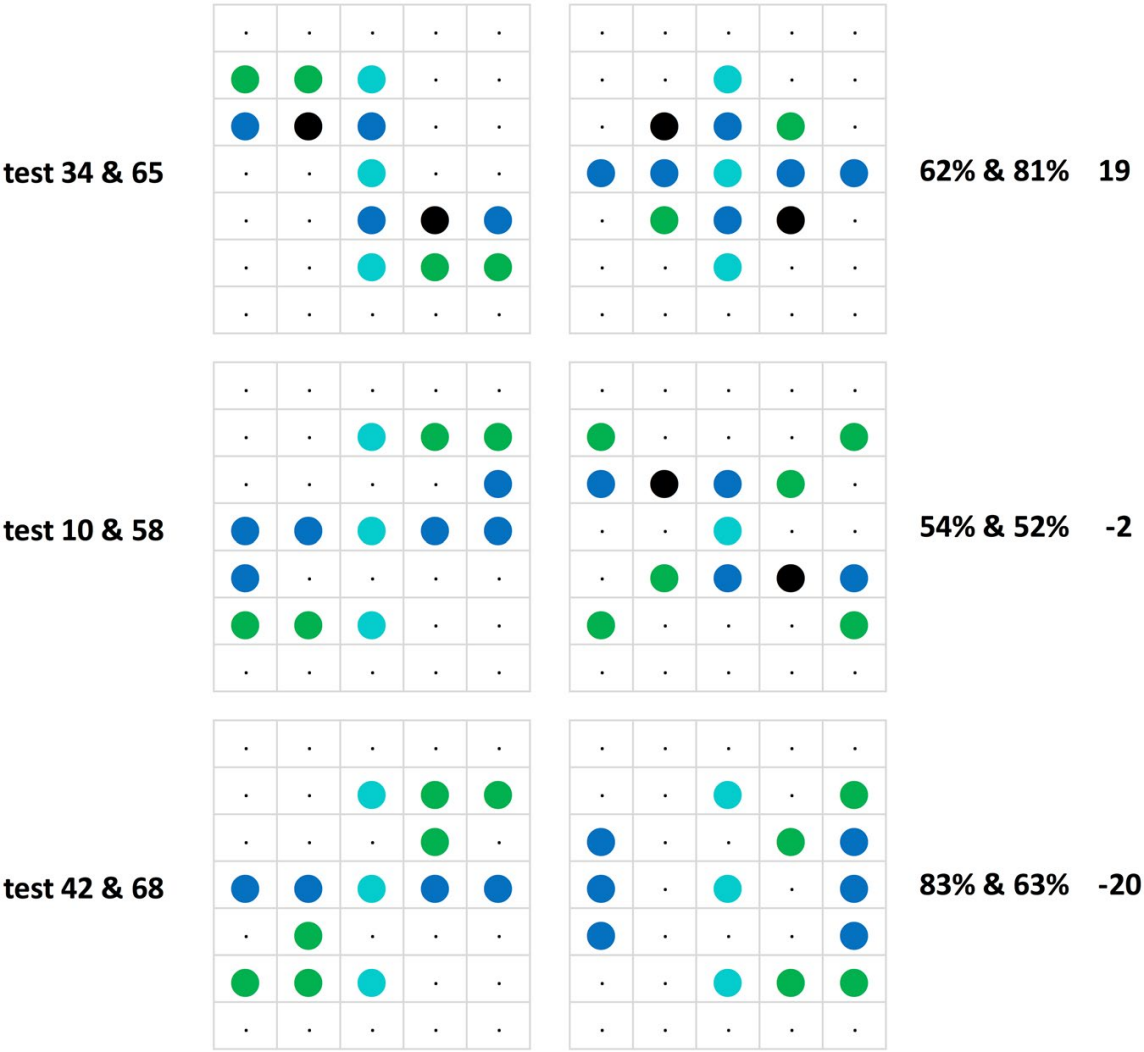
Three examples of repeated testing with the same pattern pairs illustrating an increase, a relative stability, and a decrease in percent choice accuracy. Larger black dots indicate pixels that were unlit in the training phase, but were sometimes lit in the test patterns.

We begin by examining the pigeons’ performance on the generalization stimuli by checking their consistency upon test repetitions. A total of 34 different stimulus patterns forming 17 pairs were tested twice; two of the pairs were tested thrice, adding up to a total of 19 repetitions (cf. Figs 6 and 7). According to their average percent correct choices, the six pigeons as a team always clung on to their initial test pattern preferences in these repetitions, though in a few cases barely so. Test repetitions did not lead to an increase in choice accuracy; on the contrary, the average 6-pigeon choice score on the second round, in the mean 28 tests later, was about −6.1 percent points weaker than on the first round, ranging between + 19 and −30 percent point differences from case to case, there being a non-significant, slight negative (r = −0.16, p > 0.05) correlation between the percent point choice differences and the extent of the test gaps across the 19 test repetitions.

It is thus likely that although the pigeons’ discrimination of the training Z* H* patterns had reached a ceiling, their capacity to distinguish the test Z° and H° patterns from the training Z* H* patterns continued to increase as the testing phases with the never-reinforced generalization pattern pairs progressed. This means that their disposition to generalize to deviant patterns would have tended to decrease with mounting training and overtraining^48,49^. The pigeons would have learned that non-training-type stimulus pattern pairs consistently yielded no reward and thus did not merit much discriminative effort. In other words, we would have wished our pattern generalization exploration procedure to be more stable but we do not know how this can be achieved: no fully satisfactory method seems to be on offer.

**Figure 8:**
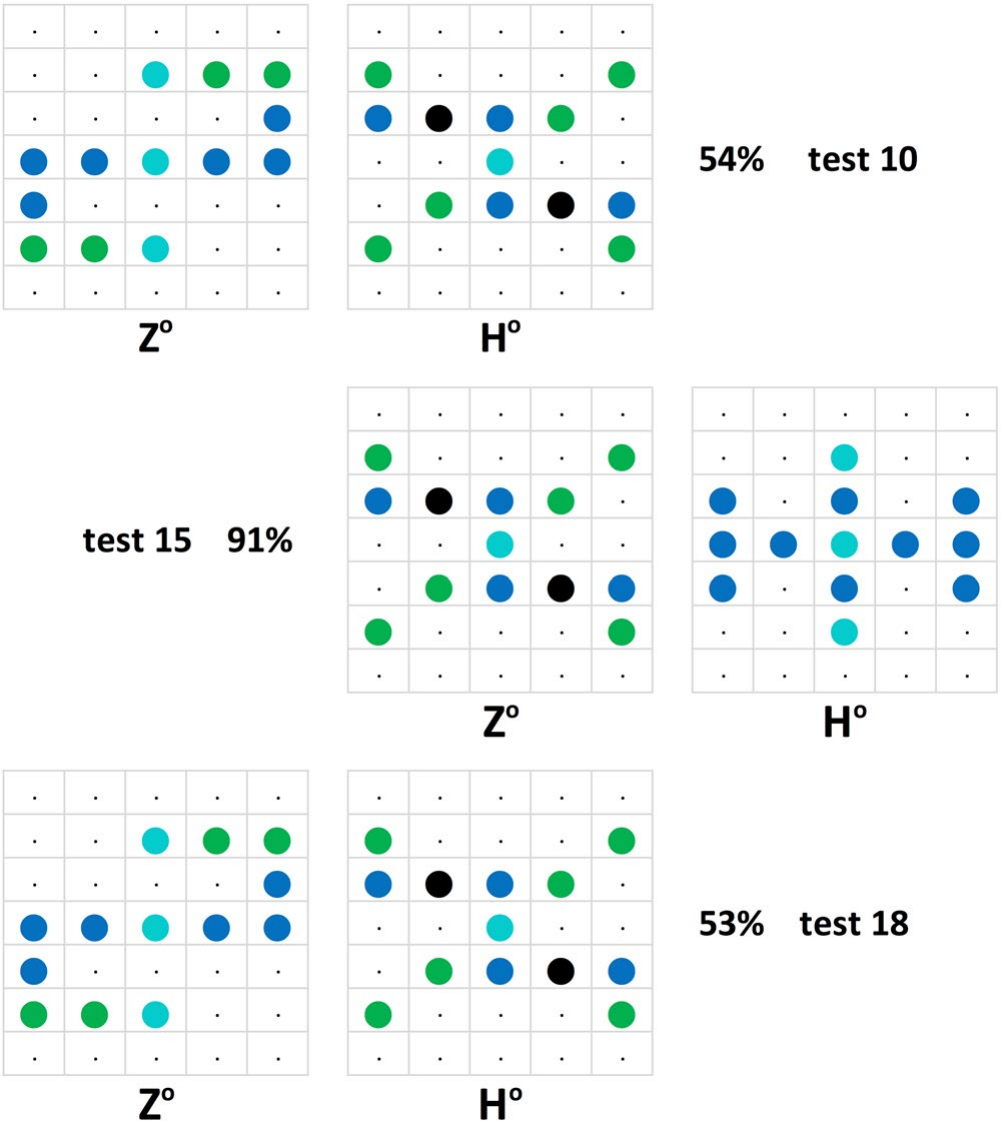
Relativity: Tests using the same stimulus pattern (resembling an elaborate letter x, middle column) paired twice with a Z-like –or rather right–left reversed s-like– stimulus pattern (left, tests 10 and 18) and once with an H-like stimulus pattern (right, test 15). The pigeons accordingly treated the middle pattern both as barely H-like (top, 54%, and bottom, 53%) and as clearly Z-like (middle, 91%).

There were also further 16 tests where given stimulus patterns were repeatedly presented, but in these instances with changing partner stimulus patterns, on average 10.9 trials later. These one-pattern part-repeat tests yielded on average −10.8 percent point less definite choice preferences with the changes ranging between + 15% and −67%. Indeed, in five instances the change in choice involved a switch from the preference for the relevant pattern to its avoidance. These marked switches came about when an intermediate Z/H°-like pattern was presented together with a patently Z°-like pattern in one test, and together with an obviously H°-like pattern in the next test, or vice-versa (Fig. 8). When the alternate patterns were not as dimorphic, the choice changes were less pronounced; regarding another instance of a similar pattern interaction see the *Translocated and compound patterns* section. There is thus a volatile relativity issue involved in our generalization evaluation scheme that still needs to be more squarely addressed.

### Pixel richness

**Figure 9:**
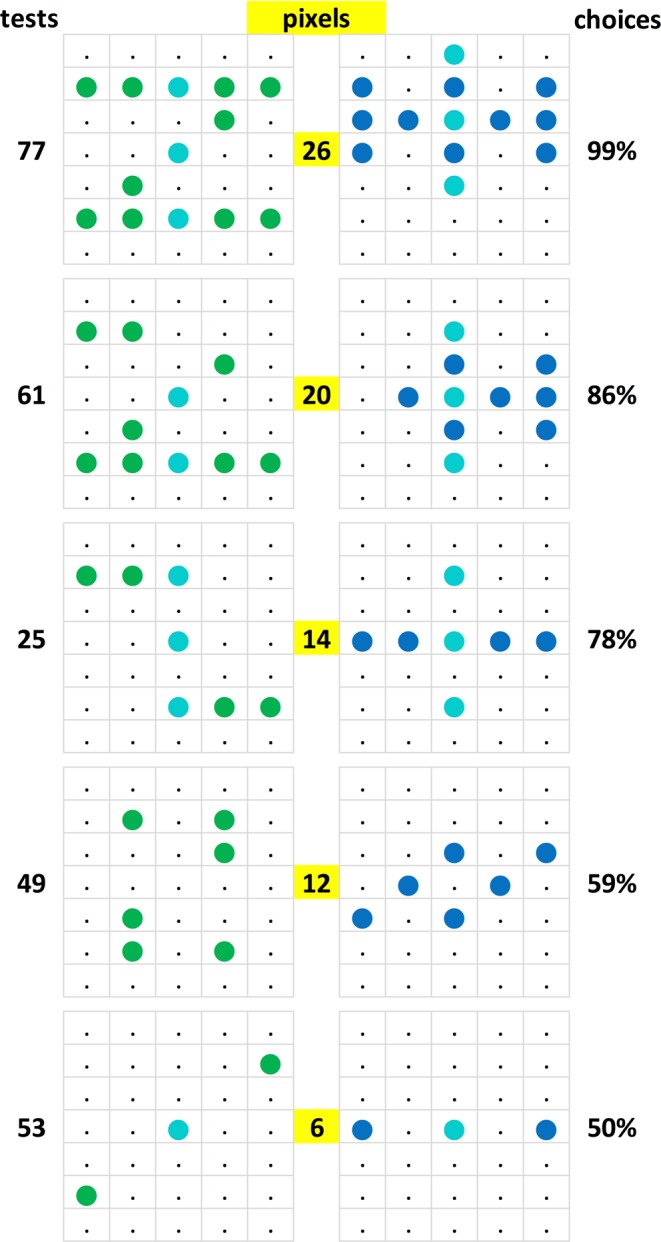
Five sample test pairs depicted in a descending order according to their number of lit pixels to illustrate the associated loss of percent pattern choice accuracy.

An obvious variable to consider regarding the discrimination of generalization pairs was the number of lit pixels that made up the stimulus patterns forming them. These counts ranged from 4–26 pixels out of the almost routinely used 50 matrix pixels and out of the total 70 existent pixels; when correlated with the average choice scores they yielded r = 0.40, n = 84, p < 0.0005. That is, the lower the number of lit pixels was, the worse on average the choice performance of the pigeons. Doubtlessly this was due to the diminishing information conducive to an adequate choice (Fig. 9). That was perhaps supplemented by the fact that as the experiment went on, the pixel-meagre pairs signalled that a discriminative effort was relatively worthless whereas the pixel-rich pairs that were more similar to the training pairs indicated that the effort was possibly worthwhile. Thus, an increasing number of relevant pixels can improve generalization but only when other variables –which we still need to identify more closely– allow it.

**Figure 10:**
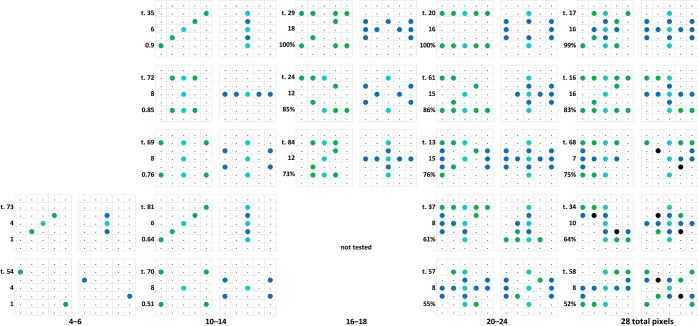
Twenty test pattern pairs, out of a total 84 such pairs, laid-out from left to right according to their ascending total pixel counts (from 6 to 28 pixels) and bottom to top according to the resulting percent choice scores (50% to 100%). The inserted numbers refer to the *Discriminability measure* of the respective pairs.

A step in that direction was the construction of a corresponding pixel count / choice accuracy correlogram (not shown here completely), which evinced a fan-like scatter pattern. Originating from the left low pixel count, no-discrimination vertex, it spread out to the right up to the high pixel counts where discriminations varied between absent to perfect. This format permitted a column-wise search for other factors affecting the choices of the patterns. Figure 10 shows a selection of 20 test stimulus pairs laid out according to their approximate position within that correlogram.

### Discriminability measure

Within the right half of Fig. 10 at least, a discriminability measure revealed itself as probably determining the respective choice scores. Even though the evidence is not overly conclusive^50–52^, it has been suggested that in learned simultaneous discrimination tasks, choices arise through the pigeons’ brain evaluating the presence of both the positive, choice-furthering and the negative, avoidance-furthering elements within the separate stimuli of a given pair. That is, the choice of the generalization test patterns here would in principle come about because the pigeons recognized the predominant presence of ‘positive’ pixels predicting reward in one stimulus and, perhaps less so, the predominant presence of ‘negative’ pixels predicting non-reward in the other stimulus.

Indeed, the measure that offered itself for ordering the various Z°H° test pairs in terms of discriminability, was the sum of numbers of lit z pixels in the Z°-like pattern plus the number of lit h pixels in the H°-like pattern, resulting in a figure ranging from 0 to 20 pixels (out of the total existent 10 z_Z_ plus 10 h_H_ pixels), disregarding the 0 to 6 ambiguous zh pixels. Across the 6 pigeons, the correlation coefficient between this discriminability measure and average choice scores was r = 0.68, n = 84 tests, p < 0.0001. This medium sized correlation supports the notion that the *z*_*Z*_ + *h*_*H*_ count characterizes the generalization value of any given pattern pair, but patently not exclusively!

**Figure 11:**
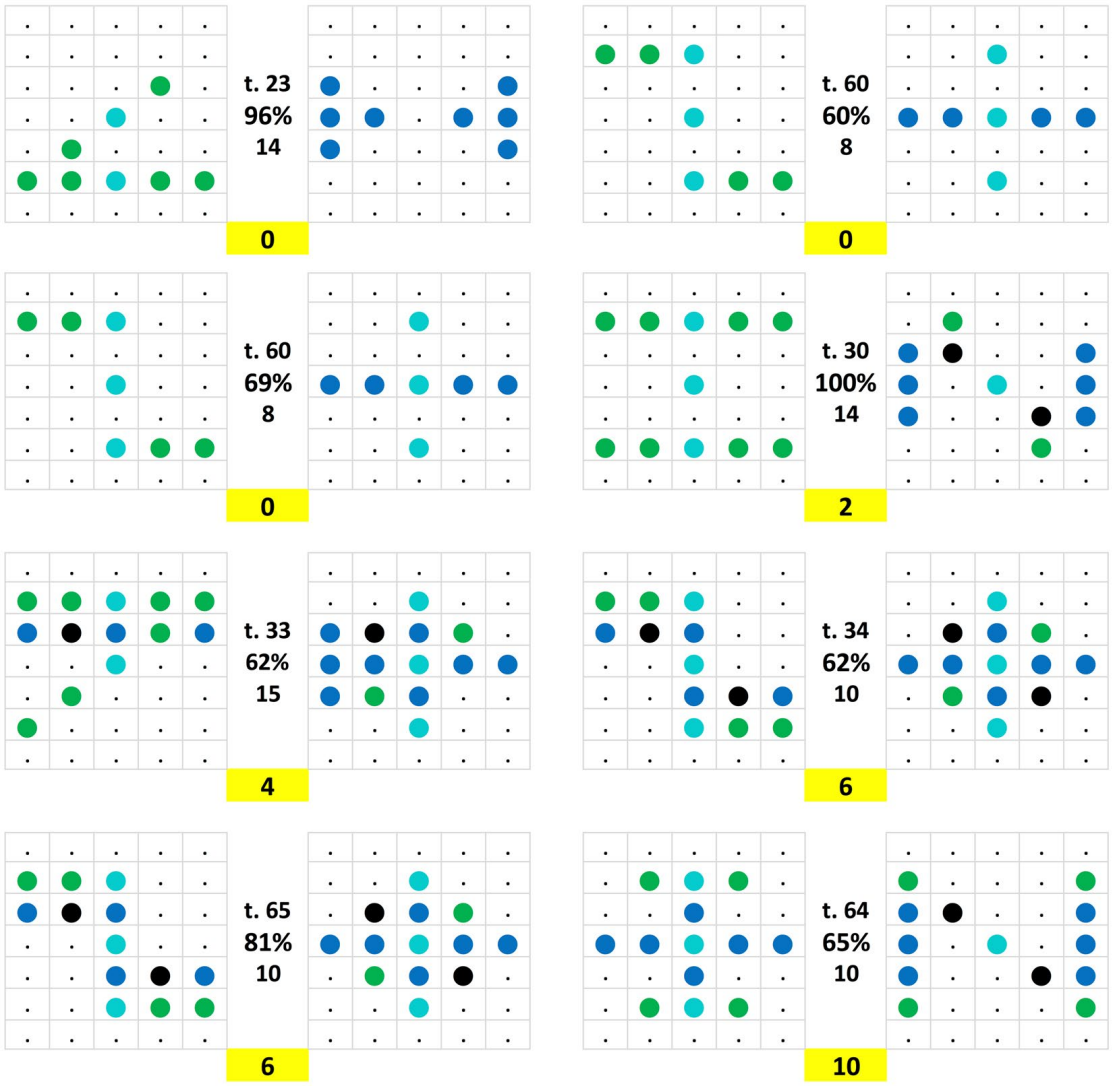
Eight test pairs forming 4 doubles ordered according to ascending (0–10) z_H_ + h_Z_ pixel counts to illustrate the on-average lower percent choice scores induced by the presence of these ‘inhibitory’ pixels. The ‘excitatory’ z_Z_ + h_H_ pixel counts (8–15) are also shown.

It also seemed possible that a subtraction of the number of h_Z_ pixels present in the Z°-like stimuli and the z_H_ pixels present in H°-like stimuli –these implying a less ‘pure’ Z°- or H°-attendant quality– might improve the discriminability measure, but in fact the h_Z_ + z_H_ count correlated negatively with the choice scores, r = −0.21, p ≈ 0.05. Accordingly, the subtraction of the count of these presumed inhibitory pixels did not lead to the expected improved overall correlation but rather led to a decrease to r = 0.48, n = 84, p < 0.01. Across 13 test pairs where the Z° and H° patterns contained no z_H_ and h_Z_ pixels and test pairs were similar, Z° and H° patterns contained between 2 and 12 z_H_ and h_Z_ lit pixels (see Fig. 11) and led to a mean 87% and 67% correct choices, respectively, the mean gap between the tests being + 2.5 tests, ranging between + 41 and −45 tests. The presence of the z_H_ and h_Z_ pixels thus yielded both a loss in choice accuracy and in association strength, that is, they occasioned a degradation in Z° and H° pattern likenesses. Surprisingly, the inhibitory elements –wrong-class pixels within given predominant-class patterns– do not further define, but rather only create a noise-like interference that affects their generalization value.

The zero to six ambiguous zh pixels that appeared lit in various test pairs could potentially have augmented the number of pattern adequate pixels (see *Pixel richness* section) by occasionally completing some distinctive Z*- or H*-like pattern feature (compare *Multipixel features* section below) but adding their number to the above difference measure only led to a very minor, non-significant increase in correlation, r = 0.69, p > 0.05. In turn, the addition of the 0 to 8 lit neutral n pixels present in some of the test pattern pairs, conversely yielded an only very small, insignificant decrease in correlation, r = 0.65, p > 0.05. These three ambiguous pixel locations thus again act principally as a weak source of noise and do not seem to contribute to the stimulus valuation via their potential feature-completing property.

### Translocated and compound patterns

**Figure 12:**
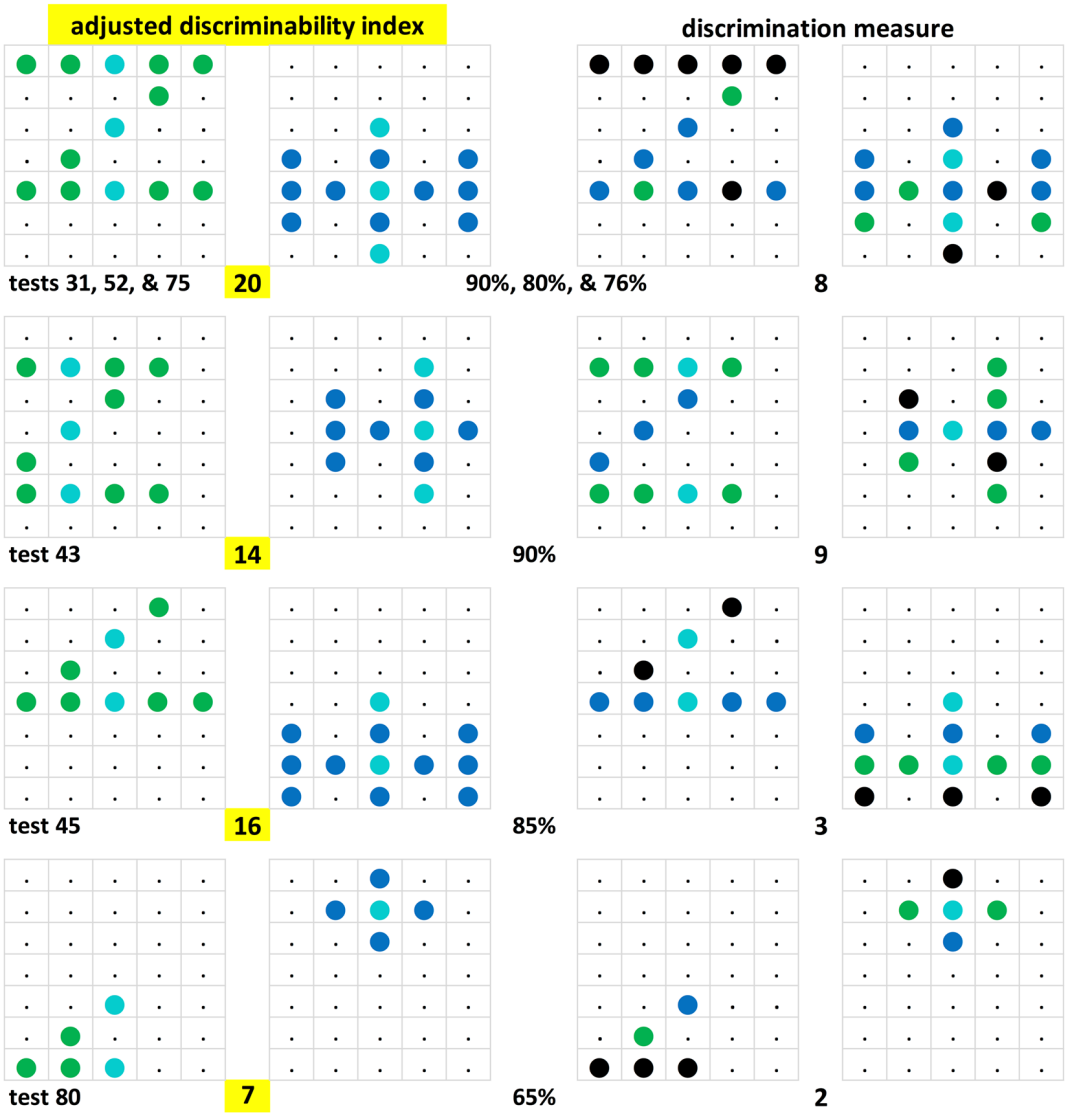
Four examples of translocated pattern pairs shown side by side with adjusted (left) and unadjusted (right) z_Z_ + h_H_ pixel counts. The rather special status of test 75 is explained in the text.

In 15 tests out of the 84 total, one or both of the Z°-like and H°-like testing patterns were shown relocated from their trained Z* and H* matrix positions. A pigeon-relative north (N) and south (S), east (E), and west (W) map-like terminology is used to describe these shifts. Note that within the 7 × 5 dimensioned matrices it was only possible to move the 5 × 5 pixel training type Z° and H° patterns by one pixel row either in the N or in the S direction; larger scale N–S translocations and all E–W translocations were necessarily associated with pixel-curtailed patterns. Four examples of such pattern translocations are shown in Fig. 12.

Based on the ample independent evidence that pigeons are prone to exhibit pronounced translational invariance during visual recognition tasks^53^, we readjusted the pixel terminology and relevant pixel counts for translocated patterns as illustrated in Fig. 12 from a matrix- (right) to a pattern- (left) centred mode and thus to a corrected discrimination index. The 17 adjustments averaged increments of 7.0 pixel counts ranging between 2 and 12 per pattern pair. In the case of tests 52 and 75 (but compare test 31), the translocations spanning two rows might have exceeded pigeons’ translational dispositions but overall, it is certain that the measures of these pairs called for an index adjustment. Unfortunately, it remains unclear whether the pigeons adjusted their viewing and pecking style to the thus apparently altered perceptual centring (see *Introduction* and *Methods*). As to test 75, note that in phase III, sessions 74 to 84, the pigeons were additionally trained with a pattern pair C formed by a Z* pattern shifted one row N and a H* pattern shifted one row S, which had no spreading effect on the pair choice performance during the accompanying tests, it quite clearly had none relating to pair 75, which involved a full four-row translation against the C training pattern pair! This large translocation appears to have exceeded the pigeons’ translational abilities, leading to a merely 76% choice correct score. But otherwise the results largely confirm that, on a smaller scale, pigeons evaluate pixel positions in a relative way. Note that it is the second time that we need to allude to such an awkward kind of relative factor.

**Figure 13:**
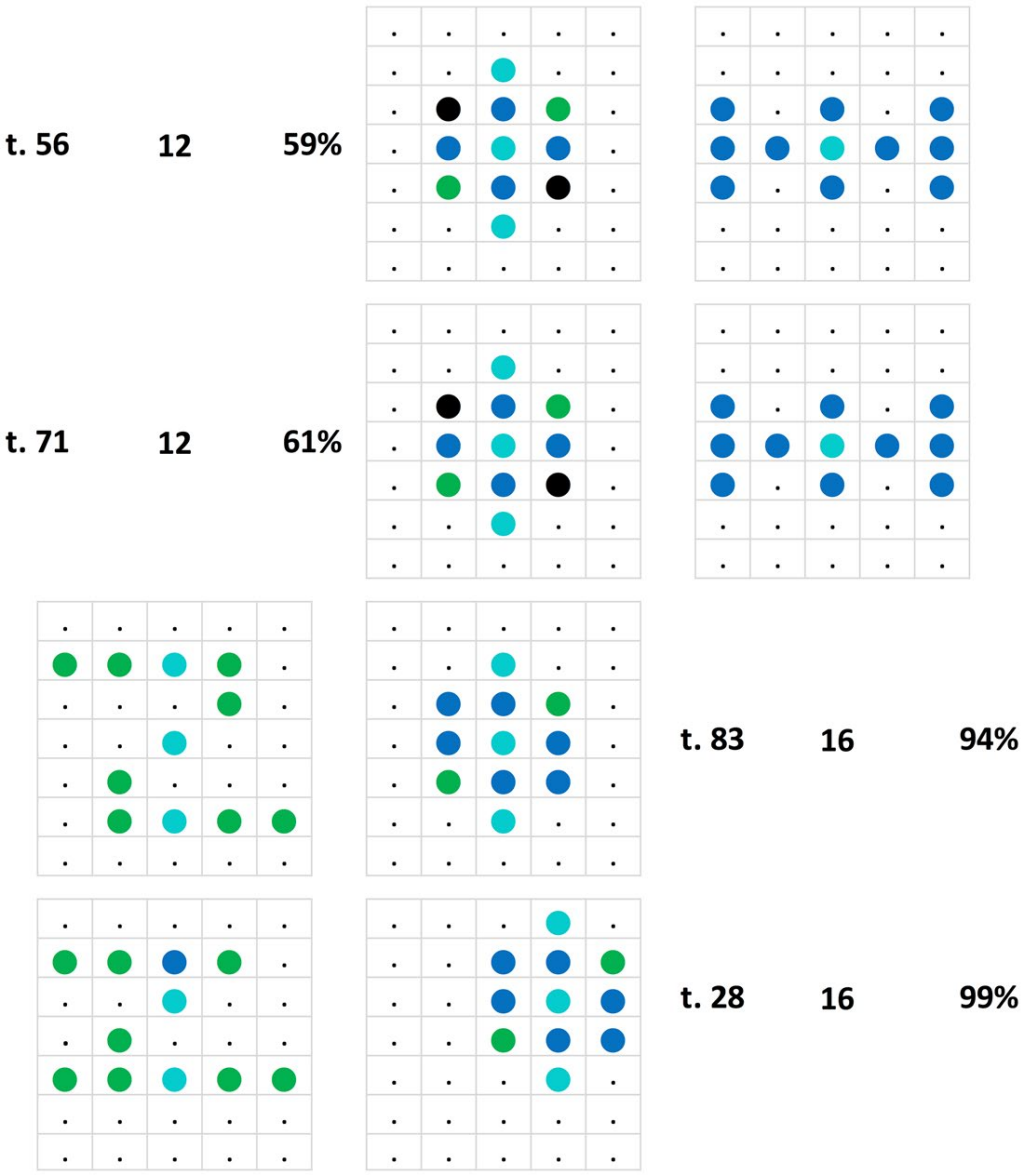
A compound test pattern (middle) was differently discrimination-assessed by the pigeons depending on whether its companion pattern was strongly Z°-like (left) or H°-like (right).

Its incidence is more directly illustrated by Fig. 13 where a given pattern was evaluated quite differently by the pigeons depending on which other pattern –H°- or Z°-like– was its pair companion. Regarding test 28 within the same figure, note additionally that the Z° pattern is assumed to have been perceived as a smaller sized Z-like pattern, pigeons having elsewhere been shown to be quite deft at size-invariant pattern recognition^54^.

Incidentally, we also presented the training pair A-equivalent with the component patterns rotated by 90° clockwise as test pairs, expecting that perhaps the pigeons would spontaneously exhibit a rotational invariance^55,56^ but they definitely did not do so. Rather, they interpreted the rotated Z° pattern as an H-like pattern and the rotated H° pattern as a Z-like pattern, thereby following the z and h pixel count principle established earlier, see test 64 (65% correct, Fig. 11; earlier identical test 32, 95% correct; see *Test replications* section, last entry in Fig. 6).

### Index/choice correlogram

**Figure 14:**
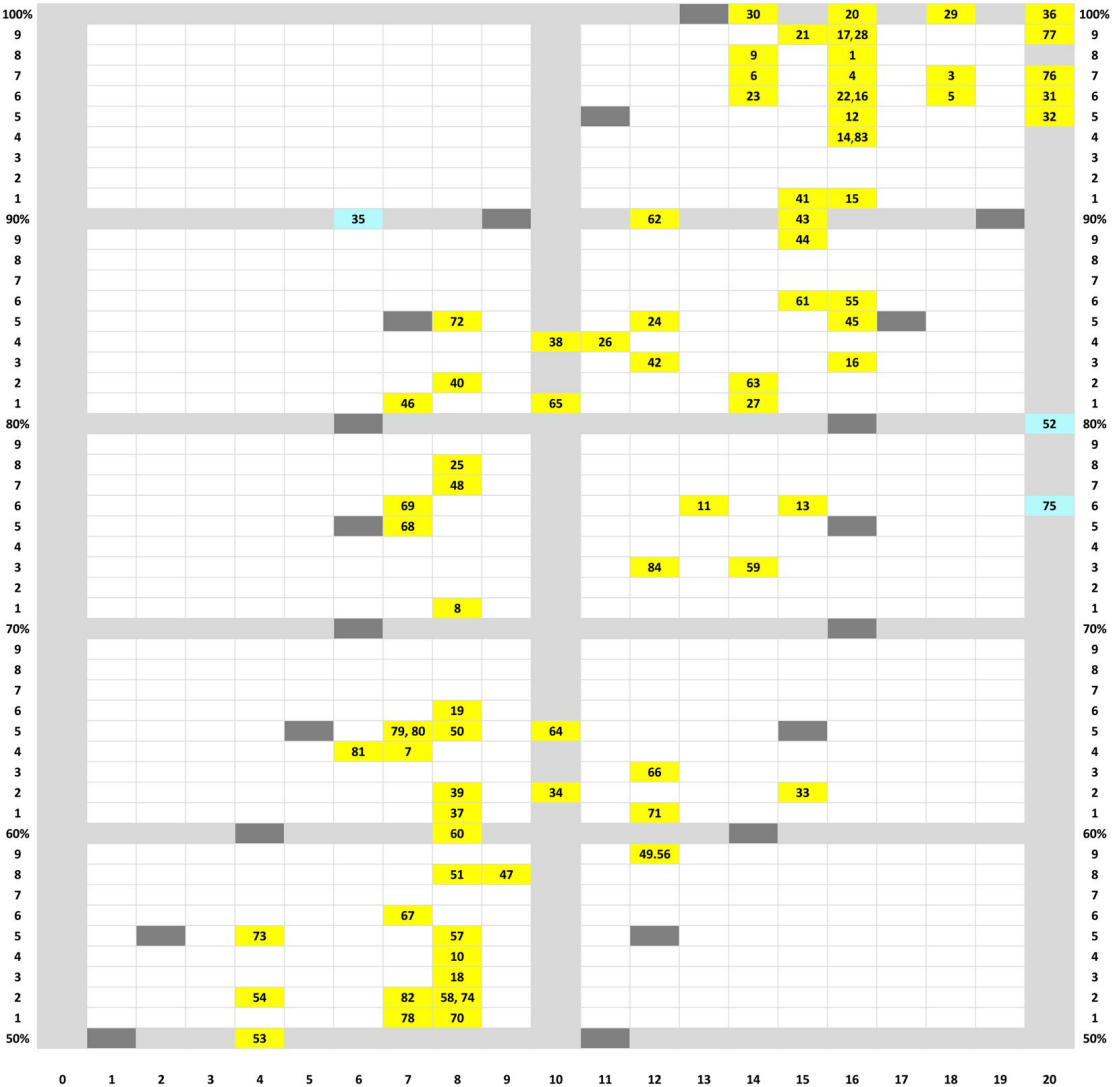
Correlogram of discrimination index and percent choice accuracy scores. Shown in grey, the ± 5 index point spreads around the midway regression line fitted by eye (but not shown). The numbers indicate tests, those falling outside this band are shown highlighted in turquoise.

Figure 14 shows the correlogram –based on corrected discrimination indices where indicated, as explained in *Translocated and compound patterns* section. It is associated with a coefficient of r = 0.74, p < 0.00001, the highest of the kind that we succeeded to obtain in this study. The regression fitted by eye is clearly sigmoid shaped. The upward curved non-linearity at low index values could hint at the operation of a mild feature effect (but compare with *Multipixel features* section below). The downward curved nonlinearity apparent at higher index values is likely to be due to a saturation effect setting in, with discriminability indices above about 15 index pixel counts.

It is striking that test pair 35 (reduced diagonal Z-like and horizontal H-like patterns), though only associated with a discrimination index of 6, evinced a high 90% choice score (Fig. 10, 10–14 column). We are inclined to attribute this to the pigeons’ relative inexperience with test pairs (see *Test replications* section) in this relatively early test 35 as the nearly identical later test pairs 67 (indexed 7, 56%) and 81 (see again Fig. 10, same column, indexed 6, 64%) yielded much lower scores.

### Individual pixel effects

Preliminary experiments using a somewhat similar procedure (see *Introduction)* had led us to expect that the pigeons’ ability to discriminate between pairs of patterns and to generalize their discriminative behaviour to novel patterns would be importantly determined by how much the patterns in question coincided or differed in terms of lit and unlit individual pixels, and less perhaps in terms of more overall features. We now necessarily revert to this notion and focus on a strictly pixel-wise analysis of the results. We began considering this by returning to the Z* and H* training patterns forming pair A, each pattern involving 13 lit and 22 unlit pixels (*Methods* section, Figs 4 and 5). The patterns differed only with regard to 20 pixels that were, by halves, alternatively lit in one pattern and unlit in the other pattern. They did not differ with respect to the remaining 15 pixels, 3 of these being identically lit and 12 pixels being identically unlit in both training patterns. Note that the three zh non-differentially lit pixels were found to play a very minor role as test pattern discrimination cues (see Fig. 17). Similarly, the two inner as well as the rarely used ten outer (rows i and vii) unlit n pixel positions –when occurring occasionally lit in test patterns– had negligible effect on their discriminability. This will be further supported in the *Discriminability measure* section below. Only the aforementioned 20 differential z and h pixel locations could thus be expected to contribute to the pigeons’ discriminative behaviour; the 3 zh and 12 n pixel locations were common to both the Z* and H* training patterns, and thus contribute little –only by occasionally completing the Z characteristic horizontal and diagonal, or the H characteristic vertical and horizontal bar elements– or nothing at all to their discriminability (but see later *Individual pigeons* section).

**Figure 15:**
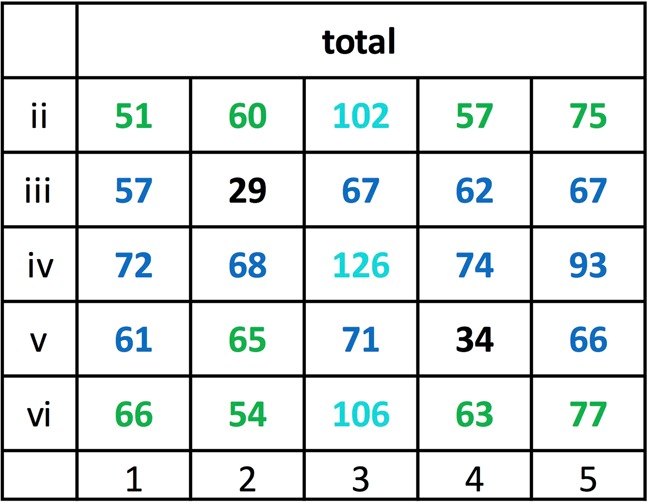
Total z, h, zh, and n lit pixel location frequencies within the testing Z°H° pattern pairs used across the 84 testing sessions. Translocation adjustments were disregarded in this compilation. The rarely used uppermost and lowermost rows i and vii have been left out (cf. Fig. 5; see also *Translocated and compound patterns* section).

As Fig. 15 shows, the 10 × 2 z and 10 × 2 h pixel locations appeared lit within a total of 2 × 84 = 168 test stimuli in between 51 and 75 test presentations. The 2 × 3 zh pixel locations were lit in 102–126 occasions while the 2 × 2 inner n pixel positions were lit only between 29 and 34 times. Note that lit z pixels were accidentally slightly less frequently present (51 to 66 counts) than lit h pixels (57 to 75 counts). This may have been partly responsible for the H^+^Z^−^ team’s overall better choice performance as compared to the Z^+^H^−^ team’s (see *Across-team consistency* section).

### Pixel-by-pixel correlations

**Figure 16:**
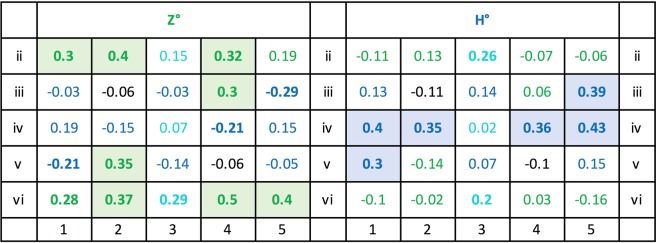
Correlation coefficients between lit pixels and the percent choice scores over the 84 generalization tests with the Z° and H° patterns and across all 6 pigeons. Coefficients ≥0.20 are printed boldface and coefficients ≥0.30 are additionally colour-shaded.

Figure 16 lists the correlation coefficients of the individual pixels of the Z°H° test pairs with the mean test percentage choice accuracies for all six subjects. It shows that 8 Z° pixel positions and 6 H° pixel positions evinced r = 0.30, p < 0.01 or higher correlation coefficients with the average correct choice scores of the 6 pigeons whereas the remainder had lesser effects. These salient pixel positions are clearly somewhat more dispersed for the Z° test patterns than for the H° test patterns by forming four clusters against only two clusters. When pooling the 1 (lit) and 0 (unlit) pixel counts within the different Z° clusters, the correlation coefficients averaged to r = 0.37, p < 0.001, within the top-left cluster; rose to r = 0.51, p < 0.00001, within the lower-right cluster; rose to r = 0.46, p < 0.0001, within the lower-left cluster; and to r = 0.53, p < 0.00001, within the top-right cluster. When pooling within the separate left and right H° pattern clusters, both correlation coefficients similarly grew to r = 0.55 and r = 0.55, ps < 0.00001. These correlations were nevertheless markedly smaller than the global r = 0.74 coefficient obtained with the overall z_Z_ + h_H_ index in the earlier *Index/choice correlogram* section.

**Figure 17:**
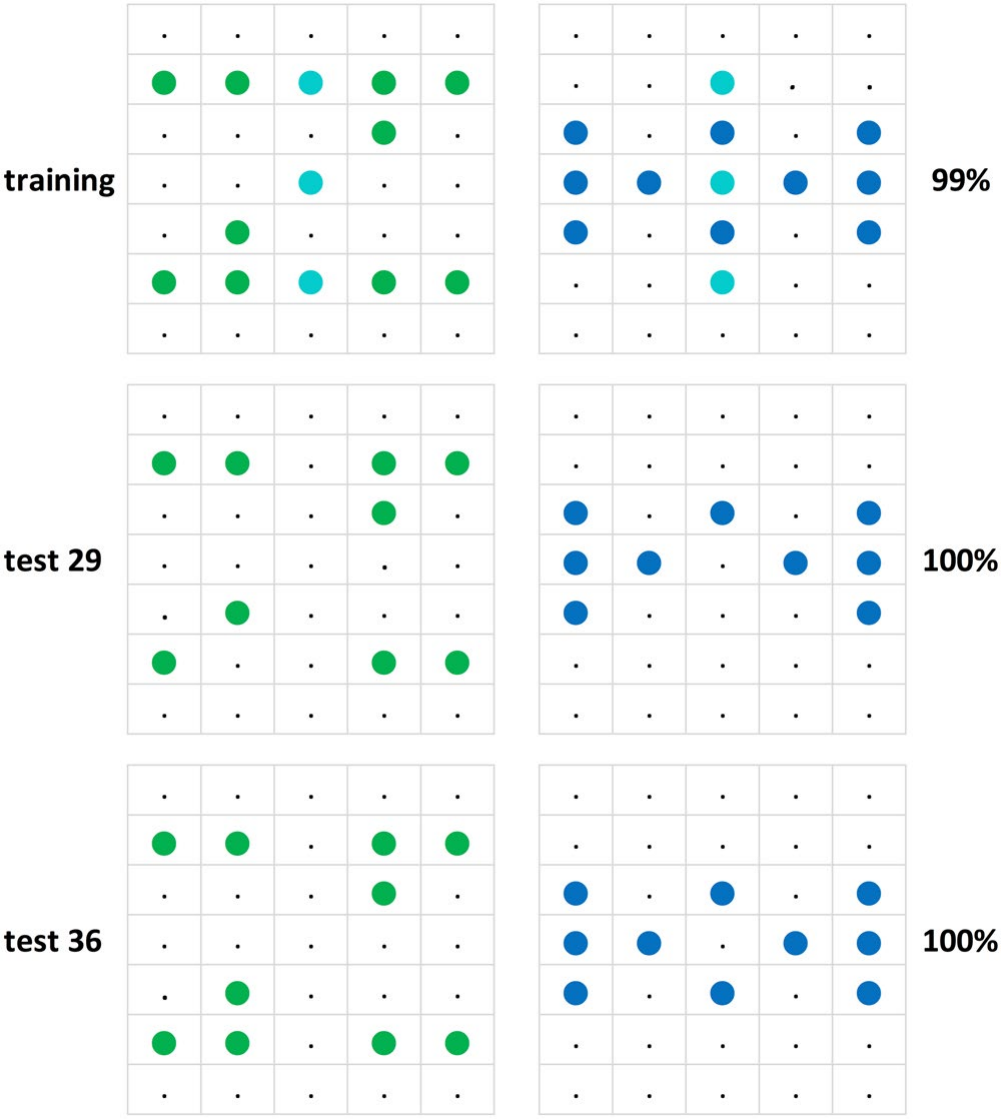
Both test 29 and test 36 pairs document the effectively absent role of the lit zh pixels for the Z°H° pattern discrimination: 100% correct test choices when missing.

As to the Z° panel of Fig. 16, its most striking detail is that the vertex ii5 and vi1 pixels are not part of two three-pixel angle clusters, features that seem so salient to the human eye within the Z° pattern. Although the pigeons were expressly trained to downgrade these two pixels between test 61 and 74 (see Fig. 4, training pair B), their relative inefficacy was already apparent in the pre-61 test results. Whether this had to do with these pixels falling outside the pigeons’ binocular field of view (cf. Fig. 3) remains uncertain: the presence of the analogously positioned ii1 and vi5 pixels in two of the other Z° clusters argues against this interpretation.

With regard to the H° panel of Fig. 16, the most striking detail is the relative non-salience of the single iii1 and v5 pixels within potential four-pixel left and right clusters respectively. It is possible that this secondary radial, rather than bilateral symmetry of the H° clusters arose as a secondary consequence of the inherently radial symmetry of the partner Z^*^ training stimulus. Apart from an enhanced sensitivity to bilateral symmetry^57^, pigeons may also exhibit some weaker generalized radial symmetry sensitivity (J. D. Delius, preliminary results). Incidentally, while ‘mind-handling’ the test pairs in connection with this paper, the senior author J.D.D. came upon the notion that the Z- and H-likeness of pixel patterns had in part to do with their respective point and bilateral symmetricity. A closer examination however revealed that for the pigeons this must have been an at most minor criterion. Viewing them at normal reading distance of about 30 cm had probably induced J.D.D. to over-rate the horizontal, diagonal, and vertical global features of the Z- and H-like patterns, whereas for the short-sighted pigeons, viewing them from about 5 cm distance (Fig. 3), and thus retinally comparatively oversized, these global features would presumably be much less salient.

Further unexpected details within the H° panel are the low correlations of both the meridian iii3 and v3 h pixel choice scores (r = 0.14 and r = 0.07, ps > 0.05). These may be due to the fact that, being geometrically single and isolated from other immediately neighbouring h pixels, the somewhat more distant, hypotenusal pixel neighbours counted less for the pigeons than the closer, cathetal pixel neighbours. This would perhaps indicate that diagonal assemblies of pixels were tendentially less feature-effective for the pigeons than comparable vertical or horizontal pixel assemblies. However, we do not have sufficient information to resolve this. In test 35 a pair made up of a partial Z° pattern consisting of a diagonal line only and a partial H° pattern consisting only of vertical lines yielded a high 90% choice score. However, in three subsequent tests (tests 46, 67, and 81) the same, though slightly translocated diagonal and vertical patterns yielded lower 81%, 56%, and 64% scores. The phase II training in sessions 62 to 74 using pattern pairs C with diagonally enhanced Z*, respectively vertically enhanced H*-like patterns, had in fact preceded the last low-score test.

The three stronger negative correlations, r = −0.29, p < 0.01, and twice, r = −0.21, p ≈ 0.05, that appear in the Z° panel while nothing like them occurs in the H° panel, are also striking. The strength of the choices of the Z-like patterns would thus appear to have been partly determined by an avoidance of h_Z_ pixels within them –puzzlingly contrary to what was more generally found earlier in the *Discriminability measure* section– whereas conversely, the choices of the H° patterns were seemingly not influenced by a similar avoidance of z_H_ pixels; but see *Pixel-by-pixel analysis* section below.

### A select index

Having identified in the previous section merely 8 z and 6 h pixel locations within 4 Z° and 2 H° clusters as being privileged, these pixels were separately evaluated for the prominent role they might play in driving the choice of the Z° or H° test patterns. Select indices were also computed according to the norms outlined in the earlier *Translocated and compound patterns* section. It consisted of the sum of the 0 to 8 z cluster pixels pertaining to the Z° pattern and the 0 to 6 h cluster pixels pertaining to the H° patterns, which accordingly varied between 0 and 14 pixels across the 84 tests. The correlation between these sum-select indices z_Z_ + h_H_ and the percent choice scores was r = 0.73, p < 0.00001, which compares quite closely to the earlier reported correlation between the overall (that is, more global) discrimination index based on all z_Z_ + h_H_ pixels and the choice score of r = 0.74 (cf. *Index/choice correlogram* section). Subtraction of the h_Z_ + z_H_ cluster pixel count pertaining to the X°Y° test pairs only had a minor detractive –rather than enhancing– effect on this correlation, r = 0.63, p > 0.01. We may thus conclude that the cluster pixels indeed played a key, although not quite exclusive, role in the test pattern choices.

**Figure 18:**
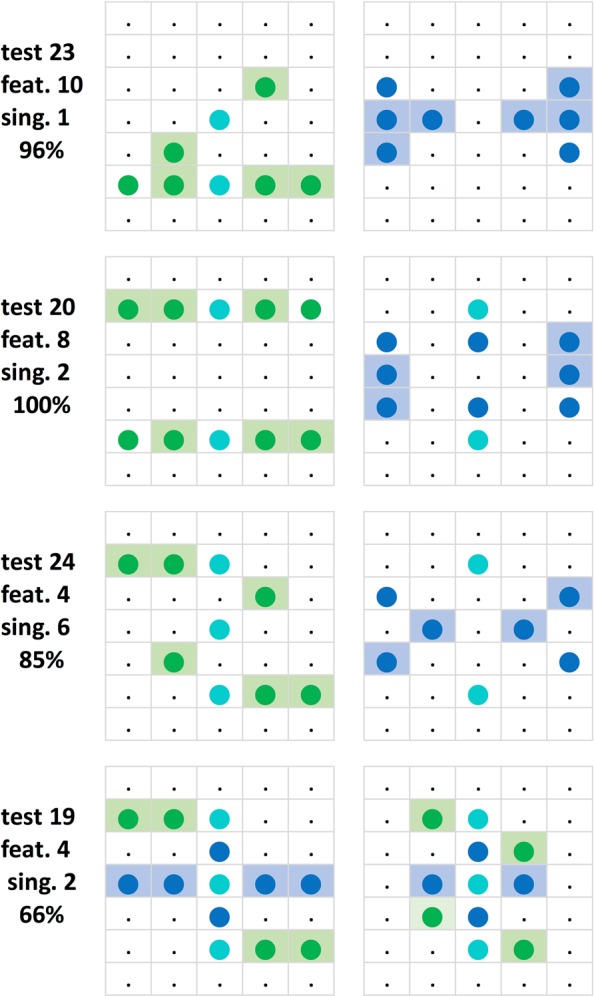
Four test pattern pairs, along with paired feature cluster pixels shaded green or blue and coloured individual single or cluster z and h pixels.

We need to return passingly to a global standpoint. Inasmuch the training patterns were distinct configurations of pixels, it is possible that the presence of corresponding arrangements of a few pixel constellations distinctive for the one or the other training pattern in the test patterns could have had an enhanced effect on pigeons’ discrimination of some test pairs, thereby perhaps occasioning the upwardly curved regression noted in the lower section of Fig. 14. Within the test patterns, we regarded any two vertically, horizontally or diagonally neighbouring conjointly lit z or h cluster pixels as a feature. Figure 18 appears to suggest that the presence of such two-pixel features might indeed boost the percent choice scores. But when we checked whether a set of 14 different such conjointly same-cluster pixels would exert a stronger influence on choice scores than another set of 14 different conjointly lit pairs of clearly non-neighbouring cluster pixels or any pair of non-cluster pixels, we found that both sets yielded quite similar average correlations, r = 0.36 and r = 0.34. This indicates that a multipixel feature ‘extraction’ was not a foremost part of the pigeons’ discrimination strategy. Perhaps this would be different if the lit LEDs constituting potential features had been less discrete and more contiguous (without void spaces between them) than they actually were in our matrices. We wonder whether it might not be wise to take into consideration a pixel-density measure in future work, that is, an actual pixel diameter / maximal pixel diameter ratio, or more simply, an inter-pixel proximity index. Incidentally, a study with human subjects^58^ who had to judge which natural scenes variously grossly pixeled images represented, had a similar aim.

### Across-team consistency

Thus far, all six pigeons were treated as forming a uniform group, despite rather patent evidence to the contrary. The two separate Z^+^H^−^ and H^+^Z^−^ three-pigeon teams constituted more homogeneous groups since their mean inter-individual test percent choice score correlations Z° mean, r_3_ = 0.83 (range 0.74–0.87, ps < 0.00001), and H° mean, r_3_ = 0.85 (range 0.80–0.87, ps < 0.00001), were markedly higher than those reported for the six-pigeon group earlier (cf. *Individual pigeons* section below).

Both the Z^+^H^−^ and H^+^Z^−^ teams exhibited concordant mean choice preferences for the Z°-like and H°-like patterns forming the test pairs in 69 of the 84 tests (binomial test, p < 0.0001). The 15 tests in which this was not the case exclusively stemmed from a subset of 30 tests in which the joint 6-pigeon average choice scores ranged only between a chance 50% and a maximally 65% preference for either the Z°-like or the H°-like pattern. The non-congruencies between the teams arose when one team exceeded the overall average choice preference for one pattern while the other team exhibited a slightly below 50% choice for the same pattern. The overall Z°-like pattern was thus doubly preferred by the two teams in 10 tests, the overall H°-like pattern being similarly doubly preferred in 5 tests. These disparities were taken into account in the analyses that follow. The low average choice scores within this subset however indicated that the Z*- and H*-like qualities of the relevant patterns were largely indefinite and that the pigeons’ choices between them were probably mainly driven by chance.

Observations suggest that while pecking at the key illuminated by the training patterns, the pigeons concentrated on one or the other illuminated individual pixel of the positive pattern for longer stretches of time, but every now and then switched to another one. When they eventually received a reward or were punished, they were thus likely to have been focusing on this or that pixel; and likewise during the test stimulus pecking that ended in neither reward nor punishment. With the same pattern they may have been reinforced one way or another for pecking quite different pixels. This course of events left room for a considerable memory updating as to the value of the different pixels based without doubt on successive overshadowing, and even on blocking processes of the kind considered by Leising, Wong, Ruprecht, and Blaisdell (2014)^59^ too elaborate to try to summarize here. But as explained in the *Methods* section we unfortunately do not have the data to explore these earlier learning developments in any such detail.

### Pixel-by-pixel analysis

**Figure 19:**
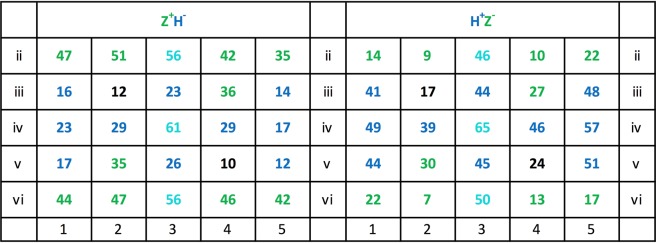
Lit pixel location frequencies within the test pattern pairs used across the 84 testing sessions segregated according to Z^+^H^−^ and H^+^Z^−^ teams. The rarely used uppermost and lowermost rows i and vii have been left out.

We now turn to the separate Z^+^H^−^ and H^+^Z^−^ pigeon teams and again to a more local level of analysis. The Z° panel of Fig. 19 shows that that the z and h pixel locations appeared lit on an average 42.5 tests, respectively 20.6 tests out of the 84 total tests, whereas the H° panel shows that the h and z pixel positions appeared lit in an average 46.4, respectively 17.1 tests out of the same total tests. Relatedly, only 7 z pixel locations out of the 10 possible ones in the Z° team test patterns against 9 h pixel locations out of 10 possible ones in the H^+^Z^−^ team test patterns accumulated more than 40 lit presentations each. This means that across the experiment, the H° test patterns were slightly more frequently present pixel-wise than the Z° test patterns, which might explain the aforementioned mildly better overall mean choice accuracy of the H^+^Z^−^ team as compared with that of the Z^+^H^−^ team. The reader should, however, be aware that few test trials were embedded among many training trials –at a 2:15 ratio– where all 13 z, 13 h, and indeed 6 zh pixels were always lit.

**Figure 20:**
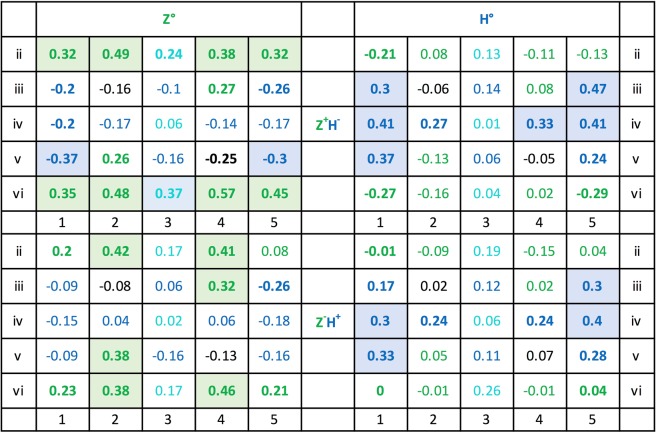
Z^+^H^−^ and H^+^Z^−^ teams, correlations of 0 (unlit) or 1 (lit) values of Z° and H° test patterns with the percent choice scores pixel by pixel i2 to v6, across the 84 tests. The coefficients ≥ 0.20 are printed boldface; those ≥ 0.30 are additionally colour-shaded.

Figure 20 further shows the 2 × 25 z and h pixel-by-pixel correlations –with the pixels valued at 1 when lit and at 0 when un-lit– with the Z^+^H^−^ and H^+^Z^−^ team test choice scores. The upper half of the table presents the Z^+^H^−^ team results; the left panel thereof depicts to what extent the diverse lit pixel locations of the Z° patterns promoted their choice (positive correlations r ≥ +0.30, p < 0.01, 8 z locations, 1 zh location) or inhibited it (negative correlations r ≤ −0.30, 2 h locations). The panel on the right shows how much the various lit pixels locations of the H° patterns promoted the avoidance of H° patterns (positive correlations, r ≥ +0.30, 6 h locations) or inhibited them (negative correlations r ≤ −0.30, 0 z locations). That is, out of 50 candidates, now 17 locations altogether –rather than only 10 as before– played an enhanced role within the Z^+^H^−^ discrimination.

The lower H^+^Z^−^ team’s left panel analogously highlights that a selection of lit z pixel locations of the Z° test patterns especially promoted Z° choice avoidance (positive correlations r ≥ +0.30, p < 0.01, 6 z locations) or inhibited them (negative correlations r ≤ −0.30, 0 h locations). The right panel similarly highlights the lit pixels of the H° test patterns that promoted H° choices (positive correlations r ≥ +0.30, 4 h locations) or inhibited them (negative correlations r ≤ −0.30, 0 z pixel locations). It thus emerges that again only ten pixel locations played an enhanced causal role within the H^+^Z^−^ discrimination. But nevertheless, this analysis differentiating the Z^+^H^−^ and H^+^Z^−^ teams shows that an overall larger pixel selection played a role than suggested by the earlier more global summary average analysis.

### Individual pigeons

**Figure 21:**
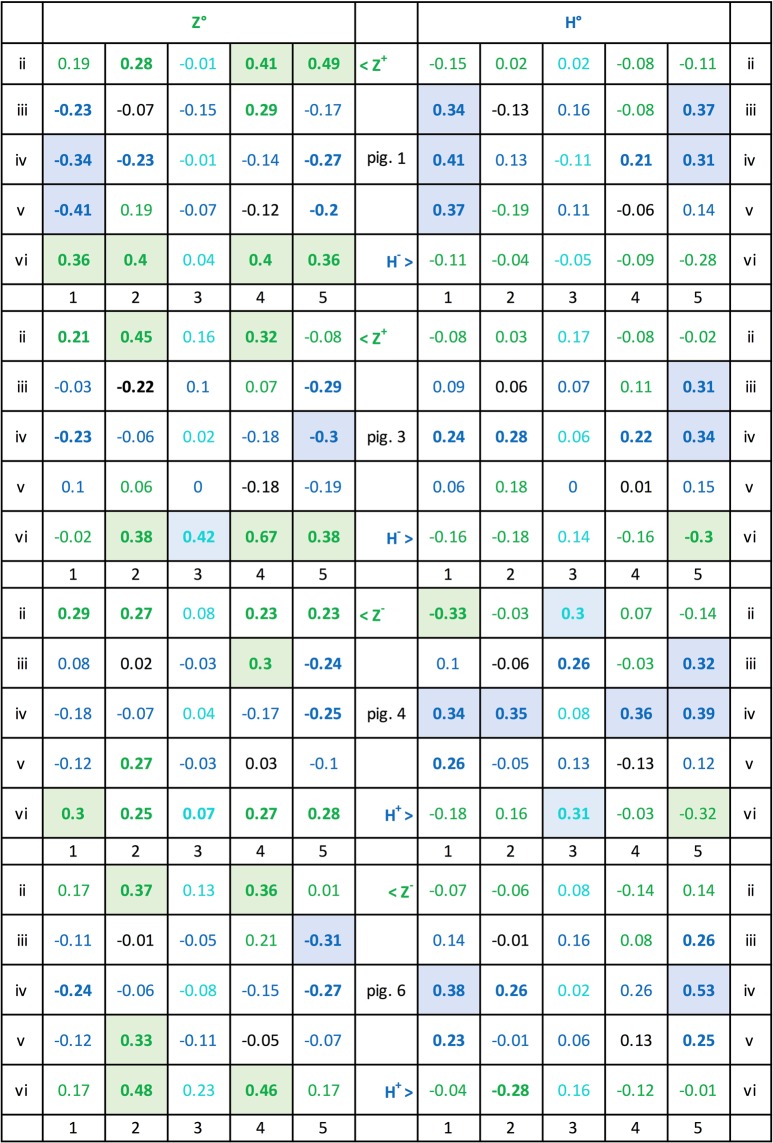
Pixel-by-pixel correlation coefficients with test choice performances of Z^+^H^−^ pigeons 1 and 3, and H^+^Z^−^ pigeons 4 and 6. Use of colour-shading and boldface as in Fig. 20.

Are the above team-wise average results representative of each of the individual pigeons or again only the result of an artefact-generating pooling? Averaging over groups of subjects is commonly viewed as a mainly measurement-error reducing device but in fact it can also serve to obscure important individual heterogeneities. In our case it must be remembered that the 6 individual pigeons’ percent choice scores on the 84 test pairs evinced 15 pairwise correlations averaging a mere r = 0.47, p < 0.00001, while ranging from r = 0.21, p ≈ 0.05, to r = 0.74, p < 0.00001. It would go too far to show the data of all six pigeons, but Fig. 21 presents four of the pigeons’ corresponding data to illustrate the issue in question. Note that the pixel-by-pixel data of each of the pigeons diverged markedly from the overall average data shown in Fig. 16 and even from the team-wise averages shown in Fig. 20. The remaining two pigeons’ data (not shown) revealed disparities of a similar order.

We can only speculate as to the origin of these inter-individual differences. Firstly, the pre-experimental histories of the pigeons were not closely controlled. Although all pigeons were trained up to the same criterion they differed in the numbers of sessions required to reach it (see *Training procedure* section). Afterwards they certainly also differed in the extent to which they required retraining, pigeon 3 (Z^+^H^−^) being somewhat notable in that respect. But we can obviously not causally attribute their varying pixel location uses to such global performance characterizations. Rather we conclude that early arising individual idiosyncrasies in peck location habits might have influenced which pixel locations were predominantly attended to. The haphazard patterns formed by the reward grains on the keys that we already referred to above might have added to the individualities by way of a conditioning noise source. The individuality of the visual brains due to differing genetic inheritance and differing developmental history must be generally reckoned with^60^.

We incidentally found no evidence of hemispheric dominance effects based on the right eye dominance due to birds’ totally crossed optic nerves and asymmetric embryonic eye development^61,62^. For example, we might have found that the left-half matrix correlation coefficients had more impact than right-half matrix correlation coefficients, but there was no sign of such an effect. Admittedly, however, we did not carry out separate analyses of trials when a given test pattern was displayed under the left or right key which might have been more incisive regarding this issue.

We did notice, however, that the south-edge, row vi correlation coefficients were slightly more impacting than north-edge, row ii ones; but this is likely to be due to the keys being spring-hinged at the north edge making them palpably easier to activate than pecks along the south edge.

## Discussion

In summary, we found that pigeons that learned to distinguish a pair of multi-pixel patterns did not do so by evenly valuing the constituent pixels that were equally informative in principle. Instead they did so by unevenly valuing some more than others, and in a manner that was largely individualistic and hard to understand. At the same time, we were not quite able to reveal the scheme according to which their brain combines these values so as to achieve the choices shown when responding to a large series of derived generalization pattern pairs. We were only able to observe that the valuing of individual patterns partially depended on which other pattern it was paired with. There may yet be other summary indices to discover –perhaps by applying multiple regression procedures yielding higher correlations than those that we have managed to uncover in this paper. Having tentatively identified promising variables, future experiments can be now designed to more systematically study their influence one by one. Generally, however, we see little future in attempts to account for the pigeons’ learned visual pattern recognition feats of this kind on the basis of a finite sequence of well-defined and well-ordered algorithmic processes that can be understood as extensions of clean classical Aristotelian logic^63,64^. Supporting this notion, Sadil *et al*. (2019)^65^ have recently shown that representations of intra-item, part-to-part associations can be learned and retrieved separately from the representation of a visual object’s identity by humans, thus demonstrating that object processing is not a pure hierarchical process. We are striving towards defining a causal framework, a neural network defined by its inter-neuronal synaptic weights that would allow us in principle to causally attribute why pixeled pattern #X yields a choice level Y% and pattern #Y yields one of Z%; only in principle, because the network might be so complex that *de facto* it can no longer be intelligibly describable (see below)!

**Figure 22:**
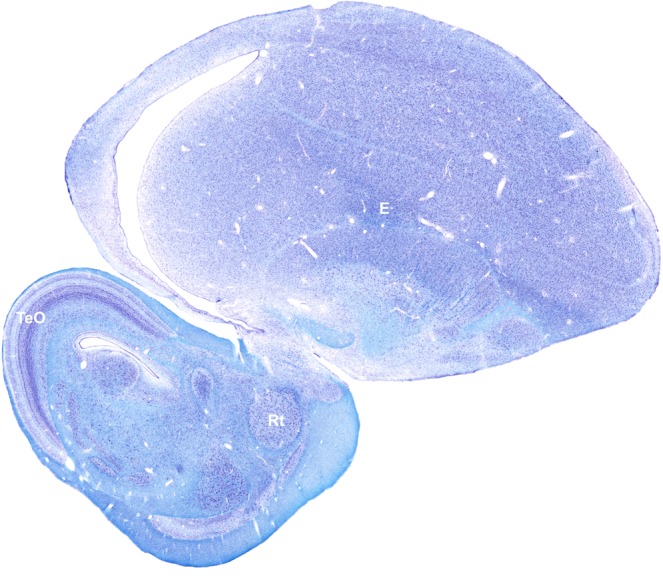
Sagittal brain-section (L3.00 mm) of a pigeon showing the tecto (TeO)–rotundal (Rt)–entopallial (E) complex constituting their principal –but by no means only– visual information processing system. Note that the entopallium is factually considerably larger than it appears in this quite medial section. Microphotograph kindly provided by Dr. Felix Ströckens and Prof. Onur Güntürkün, Bochum.

In a kind of extension of Tsien’s (2015)^66^ arguments about neuronal nets, we turn to the close to infinitely complex network functioning of (avian) brains instead (see Fig. 22). The optic nerves of birds, already originating in a neurally comparatively rich retina terminate in the optic tecta (TeO) of the mesencephalon, which in turn project mainly to the diencephalic nucleus rotundus (Rt)^67^, which again projects to the voluminous entopallium (E, former ektostriatum) of the telencephalon. It is reasonable to expect that the main stages of visual pattern recognition of birds take place there; the retino-thalamic-telencephalic projection system, so prominent in mammals, is far less prominent in birds^68^. Although in previous times the midbrain was assumed to handle innate responses to stimuli such as avian neonatals’ preferential pecking at certain coloured sticks or dots (pigeons^69^, gulls^70^), these days it is thought to be the substrate of conditioned visual responses thanks to the profusion of biochemical markers known to be symptomatic of synapses modifiable by learning. Avian anatomy and physiology establish the optic midbrain as a natural neuronal network of mega-dimensions –the tectum opticum alone has some 15 neural layers– embodying about 100.000 retinal, 10.000.000 tectal neurons, again 1.000.000 rotundal, and 10.000.000 entopallial neurons) inter-connected by perhaps 1.000.000.000.000 synapses, three quarters of which are likely to be modifiable through learning^71^. The network must extend neurally further ‘down’ to reach the 100.000 or so spinal motoneurons orchestrating the 20 or so vertebral and cranial muscles activated during every peck^72^. It needs to be remembered that the pigeon’s forebrain neuron density (neurons per cubic millimetre of tissue) is about six times higher than in similar sized mammals^68,73^, large crania being disadvantageous for flying. Bird brains are furthermore particularly interesting because they harbour quite massive visual feedback, neural connections from the post-tectal isthmo-optic nucleus to the retinal ganglion cells^68^. Such recurrent connectivity is attracting much interest in the field of artificial neural networks because of its crucial role in temporal pattern (= behaviour) recognition.

Notably, the psychological relevance of –admittedly far too simple– neuronal networks was already well recognized in the 1950s, but then prematurely dismissed because of their (apparent) shortcomings due to their initial simplicity. Meanwhile it has been shown that artificial multilayer neuronal networks consisting of far fewer neuromimes and synaptomimes than real neurons and synapses are already capable of mighty visual processing feats that they can easily be trained to perform via procedures resembling perceptual learning. Current examples thereof are commercially available autonomous lawnmowers, vacuum cleaners, and observation drones ‘brained’ by such networks. We are told that the quite complex connectivity patterns that the individual gadgets’ networks develop by training themselves to mow the same lawn (or whatever their task), can be quite different though they may perform quite comparably (compare *Individual pigeons* section above). For in-depth discussion of a perceptual learning feat by an organismal neuronal net, see^74,75^; for an easily intelligible introduction to technical neural networks, see^76^; and for a more advanced overview, see^77^. While such so-called deep learning used to be dependent on a trial-by-trial error backpropagation scheme unlikely in real neural systems (which allow no retrograde transmission!), there are more modern, alternative schemes where learning of a similar kind is brought about by straightforward failure or success feedback schemes analogous to those acting in reinforcement-based organismal conditioning^63,78,79^. They may even have an advantage because they are less likely to end up being trapped in local conditioning optima. Moreover, they be may be more efficient for networks operating in dynamically ever-changing environments, which need to be continuously adapted to rather than being finally learned.

An instructive example of a complex artificial network of practical application is described by Januszewsky *et al*.^80^. The paper deals precisely with the elucidation of the connectivity within neural tissue through application of artificial vision techniques based on serial electron-micrographs of the avian brain portions. An avian visual system, which is several orders of magnitude more complex than these artificial networks, can thus be reckoned to be capable of human mind (= brain)-boggling cognitive feats. The resolved pixel stimuli used here could be useful in connection with the now feasible miniaturized life brain microscopy^81,82^ (for a bird-song recognition effort based on a neuronal net, see^83^). The pixeled patterns used here might be helpful in post-fact tracking of how different patterns are recorded in nets through the formation of differing connectivities. Of course, they will still be far from the angular resolution achieved by the retinal receptors of pigeons that is close to that of humans^84^. Note though that the linear acuity of pigeons is much superior due to their extreme myopia^42,85^. Further, if one taught pigeons to discriminate many different pairs of patterns generated using the same 5 × 7 light diode matrices, would their visual net begin to show any signs of informational saturation^86^?

One could begin to investigate whether artificial neural networks would mimic the pattern recognition performance of our pigeons, and of course those of many other visual cognition feats by many other pigeons in our and other laboratories. Perhaps in our specific case, one could start by programming a network with an input of a 30 × 20 planar element input array (allowing for the sensing of all the LEDs, the spaces between, and the edges around them) and some 10 layers of neuromimes interconnected with variable, conditionable synaptomimes, perhaps some 100.000 of them laid-out to be capable of so-called deep learning and ending up in a pre-output layer incorporating fixed mutual inhibiting motoric connections leading to in a left or right output corresponding to the two responses to the keys of the conditioning platform. We think our purely discrete-elements-type stimuli with the attending correlative analysis we have employed may be useful when attempting to track down and correct suboptimal perceptual performances of given deep learning neuronal nets simulations through modifications of synaptic weight adjustment operators and/or adoption of differing neural connectivity schemes. In the extreme case, one might need to apply a brute-force evolutionary scheme based on mutation and selection to hit upon the appropriate optimal network solution^87^. Rather than employing a non-neural error backpropagation scheme one would want to employ a reinforcement-based evaluation loop widespreadly providing feedback to the connecting synaptomimes on how good or bad the outcome of an action was, thus emulating the action of the ‘self-stimulation’ (= reinforcing) system^88^. This may create a connectivity architecture, that is, a connectome that could resemble the functioning and performance of the real brain of pigeons faced with the present pixel pattern recognition task^89^. However, these studies show that the neural network approach demands considerable special computational-methodological knowledge and skills so that we must necessarily leave them to others. Our data sheets are all available in an open-access repository for this purpose (see below).

## Conclusion

The learned recognition of medium-complex small discretely pixelated patterns by pigeons was analysed with experimental probes and found to be controlled by a complex selection of factors in a presumably highly interactive way that was additionally inter-individually different. It is surmised that this was the result of deep learning processing in myriad-sized neuron networks associated with the retino-mesencephalic-thalamic-telencephalic visual system of pigeons (birds generally) that is best modelled with preformed neuronal network computing models. In brief, we assume that pigeons exhibit no more than a variant of artificial intelligence, namely a natural intelligence ‘engineered’ by natural selection and conditioned by individual experience.

## Data Availability

To enable the modelling suggested above, tables of average, team-by-team, pigeon-by-pigeon, test-by-test, stimulus-by-stimulus, pixel-by-pixel, and percent correct choices as well as an explanatory text are deposited under: Delius, J. D. & Delius, J. A. M. Pigeons’ pixel pattern recognition. *Open Science Framework*. 10.17605/OSF.IO/MKFA5 (2019).
